# Motivations, experiences and perspectives of physicians in Germany regarding monitoring routines in coronary heart disease and post-stroke patients: A qualitative interview study

**DOI:** 10.1371/journal.pone.0348530

**Published:** 2026-09-11

**Authors:** Julia Heisig, Laura Rink, Carolin Gonschorek, Lydia König, Lisette Warkentin, Susann Hueber, Thomas Kühlein, Annika Viniol, Veronika van der Wardt

**Affiliations:** 1 Department of Primary Care, University of Marburg, Marburg, Germany; 2 Institute of General Practice, Friedrich-Alexander-Universität Erlangen-Nürnberg (FAU), Erlangen, Germany; PLOS: Public Library of Science, UNITED KINGDOM OF GREAT BRITAIN AND NORTHERN IRELAND

## Abstract

Chronic conditions, like coronary heart disease and post stroke status require lifelong care which negatively impacts upon patients’ quality of life and increases burden on the healthcare system. Monitoring routines in chronic conditions, including echocardiography and carotid ultrasound, should have evidence of benefit for the patient, detect deterioration and guide treatment adaptations. However, monitoring routines often lack evidence for intervals, specific tests or apparative diagnostic tools. The aim of this qualitative interview study was to identify the motivations, typical procedures and consequences of monitoring including echocardiography or carotid ultrasound in patients with coronary heart disease or stroke. We conducted semi-structured interviews with 22 physicians from three different specialties (general practitioners, cardiologists and neurologists) in two German states. Transcripts were analysed with thematic analysis. Physicians considered monitoring generally as beneficial for the patient and the health system, as it helps to detect and in consequence prevent disease progression through adaptation of therapy. Physicians discussed the risks of monitoring like overdiagnosis, overuse and overtreatment when examinations are done too frequently. Reasons and motivations given for echocardiography on coronary heart disease patients and carotid ultrasound monitoring on stroke patients were diverse and ranged from individual feelings and values, like personal assurance and safety concerns for patients, to external factors like financial incentives. Ultrasound examinations as routine monitoring procedures were considered as controversial. Physicians reported insufficient medical benefit for the patient and rarely saw therapeutic consequences. These regularly performed ultrasound examinations strain resources in daily practice and limit availability for acute care. In order to achieve a more efficient and balanced use of monitoring examinations, systematic changes are required at a political level. Further research is needed to evaluate monitoring routines and incorporate evidence-based recommendations into guidelines that optimize patient care and resource use.

## Introduction

Chronic disease is defined as a condition of long duration (3 months or one year and longer) that cannot be cured and patients are dependent on lifelong care or treatment [1]. Chronic conditions like cardiovascular diseases, and post stroke status, chronic respiratory diseases and diabetes place a burden on both patients’ well-being and quality of life as well as the healthcare system [2,3]. Patients with a chronic condition are regularly examined to manage and optimize the treatment for their long-term health problem and to check on progress or regress of the disease [4]. Other conditions like cancer, depression, osteoporosis or arthritis also require regular medical observation and treatment. Typical monitoring routines of chronic conditions are conducted regularly independent of complaints or other findings [4].

In Germany, the ambulatory and hospital sector are separated in terms of organization, financing and reimbursement [5]. The ambulatory care includes general practices as well as other self-employed outpatient practices of medical specialists. Patients can see those directly, without referrals from general practitioners (GPs). GPs or other specialists (e.g., cardiologists, neurologists, dentists, midwives) are office-based in single or group practices and operate largely independently from hospitals and from each other. General practitioners are typically reimbursed by statutory health insurers on a fee-for-service basis, with most services billable only once per patient per quarter [5]. For some chronic diseases, patients can enrol with their health insurance provider to take part in a structured disease management program (DMP) [6]. This ensures that they are treated according to the current state of medical knowledge across the boundaries of individual healthcare facilities. Monitoring within the DMP is performed by physicians and ranges from questions about symptoms, function or quality of life to simple physiological tests and technically complex or invasive measures [4].

Monitoring should be beneficial for the patient [4,7]. Physicians assess the progress of disease, adjust treatment, check if the treatment is right and detect early complications [8]. Despite financial costs and emotional commitment from the patient, monitoring often lacks evidence for its benefit and harm [7]. Guidelines often give expert-based recommendations that lack evidence in terms of intervals or specific tests or specific apparative diagnostic tools [9]. Recommendations are consensus based as studies on advantages of specific intervals or on evaluating follow-up assessments of monitoring are missing [10,11]. When monitoring guidelines are vague or not well evidence-based, this can create uncertainty for clinicians and lead to further diagnostic testing that may not be necessary [12].

This interview study was part of a project that aims to formulate recommendations for evaluations of **Mo**nitoring routines in patients with **Chro**nic conditions (ChroMo). One subproject of ChroMo involved a health services analysis aimed at providing an overview of existing monitoring practices within the German healthcare system (routine data analysis and interview study). Another subproject focused on developing recommendations for the scientific evaluation of monitoring measures in the healthcare system, comprising a scoping review, a systematic survey of clinical guidelines [12], expert panel–based development of methodological recommendations, and the formulation of health and research policy recommendations. The objective of the interview study was to analyse the current state of monitoring routines and exploring physicians’ and patients’ rationales and views. As two examples for monitoring strategies and as specific topics for discussion during the interviews with physicians, we chose monitoring using echocardiography in patients diagnosed with coronary heart disease (CHD) and monitoring with carotid ultrasound in patients after stroke. CHD [11,13] and stroke [14–16] guidelines offer limited evidence-based recommendations for routine imaging follow-up including ultrasounds. These examinations are regularly performed in Germany despite being explicitly not recommended for patients with uncomplicated CHD and lacking clear evidence of benefit [11]. This paper focuses on interviews with physicians. A separate paper from the ChroMo study, presenting patients’ views and experiences with their chronic condition and ultrasound monitoring, was recently published [17].

Within the ChroMo project, this paper contributes a multi-perspective understanding of why monitoring practices continue despite limited evidence, combining physicians’ and patients’ interview findings as well as routine data from the broader project. It links the lack of evidence base with clinical practice behaviour and perceived benefits across different stakeholder perspectives. By including GPs, cardiologists and neurologists, and by focusing on two clinical examples, the study highlights how clinical reasoning, perceived patient needs, safety concerns, collaboration patterns and reimbursement structures interact in everyday monitoring practices.

The aim of this paper was to identify the physicians’ perspective of the current situation of monitoring in the German primary care health system based on two examples of chronic conditions. The research question for this study was: How do GPs, cardiologists and neurologists describe their motivations, typical procedures and consequences regarding monitoring with echocardiography/carotid ultrasound of patients with CHD/after stroke?

## Methods

### Study design

We conducted semi-structured interviews with GPs, cardiologists and neurologists to explore our research question in depth with the relevant medical specialists. We followed the COmprehensive consolidated criteria for REporting Qualitative research (COREQ) [18]. See S1 Table for the COREQ checklist. The standard of quality for this study was reflected according to Stenfors et al. (2020) [19].

The study was approved by two local ethics committees (University of Marburg, 17 May 2023, ref. 23–94 BO and University of Erlangen-Nürnberg, 12 June 2023, ref. 23–204-Bn). The study was conducted in compliance with the Helsinki Declaration [20]. We obtained formal written consent from all participants.

### Study sample

Participants were recruited by two study centres of University of Marburg in Hesse and University of Erlangen in Bavaria, Germany. The recruitment period for this study was 20 June 2023–22 March 2024. Invitations for GPs to participate were sent out via email through the research practice network in Northern- and Middle-Hesse and Northern Bavaria. GPs were also approached and personally invited at institute events (Primary Care Day, Erlangen/Bavaria, Germany). Cardiologists and neurologists in Hesse and Bavaria were invited personally to participate via email, with addresses obtained from the practices’ websites. Cardiologists and neurologists from the local region were initially approached; if no response was received within a few days, recruitment was expanded to a broader geographic area within Northern- and Middle-Hesse and Northern Bavaria. Recruitment efforts were finalised when all participants appeared well-balanced regarding practice setting (region, rural/urban and single/joint), specialty, age and practical experience. We planned to conduct 15–25 interviews to ensure that all three professional groups were represented and that a reasonable range of perspectives and experiences could be captured in proportion to the overall study design.

Interested physicians contacted the study centre in Marburg or Erlangen by phone or email. We used purposive sampling by selecting a comparable number of physicians from both study sites, from rural and urban regions, of all genders and a wide age range. Interviewed physicians received a payment of 100 € as participation compensation.

### Data collection

The development of the semi-structured interview guide was informed by existing literature on clinical monitoring [7,8] combined with the perspectives of clinical academics involved in the project (GPs and co-authors). This resulted in a qualitative design grounded in both conceptual and clinical expertise. The interview guide (S2 Table) was developed in a multidisciplinary team (with backgrounds in medicine, health sciences, psychology, and biology) and underwent extended discussion in the departmental qualitative research workgroups. The interview guide was pretested twice by the two interviewers (LR, JH) and two GPs (one was a female GP with several years of work experience who had recently started as a research associate at the Institute of General Practice, University Erlangen; the other was a male GP and professor at the Department of Primary Care, University Marburg).

The main topics of the interview guide were the importance of monitoring in general, monitoring routines of a fictional patient case (either with CHD or after stroke), motivations and reasons for specific monitoring examinations, collaboration with other physicians and patients, and consequences of monitoring (S2 Table). Half of the GPs were interviewed using the CHD case, and the other half using the stroke case. Cardiologists discussed the CHD case, whereas neurologists discussed the stroke case.

Authors JH and LR conducted the interviews, either in person in the physicians’ practices or per video call (https://docs.bigbluebutton.org/ or https://www.zoom.com/). JH and LR were both female research associates with experience in qualitative interviews. JH had a PhD in Biology and 6 years’ experience in primary care research, LR had a MA in Sociology, experience in medical market research and 3 years’ experience in primary care research. The interviewers did not know the participants prior to the study. The interviewers made it clear before the interview that they do not have a vocational education in the medical field and that they might ask more questions where medical knowledge is required. JH conducted the interviews with cardiologists and GPs with focus on patients with CHD; LR conducted the interviews with neurologists and GPs with focus on patients after stroke. Only the interviewer and the interviewee were present at the conversations. Field notes were made directly after the conduction of interviews. Physicians were interviewed until new interviews did not lead to new insights and themes, and the interviewers as well as the research team felt that sufficient data had been collected to answer the study questions. Given the broad study aim, the diversity of participants, and our intention to capture a wide range of perspectives and experiences, we decided to conduct more than the minimum number of interviews, even though the strength of the interview dialogue (supported by well-trained and experienced interviewers) would also have justified a smaller sample [21].

Interviews were audio recorded, pseudonymised and subsequently transcribed verbatim by an external professional transcription service [22]. Demographic data of the participants (gender, age, specialty, vocational experience and practice characteristics) were collected with a custom-made questionnaire.

### Data analysis

Data were managed in MAXQDA (2022) [23]. Two authors (JH, LR) coded the interviews independently and discussed the coding process regularly with colleagues from the ChroMo team to receive feedback and resolve disagreements. Analysis was carried out following the reflexive thematic analysis according to Braun and Clarke [24–26]. Benefits of this method is its flexibility and the possibility to obtain a rich and detailed, yet complex account of the material. Despite its absence of a clear and concise guideline, thematic analysis advises to follow six steps [24].

The first step for the researchers was to familiarize themselves with the data by listening to the audio recordings, reading the transcripts and taking notes. Some transcripts were read out loud by two speakers to a group of other experienced qualitative researchers from our Institutes (Institute of Primary Care, University Erlangen and Department of General Practice, University Marburg). Notes were made and discussed in the group. In a second step, relevant text passages were identified and assigned to first codes, then organized in groups. The third step was to summarize and collate the codes to generate higher-level themes. Themes were generated from the data and participants answers (inductive approach) and from the interview guideline questions (deductive approach). The fourth step was to refine themes and build subthemes until the themes outlined/encompassed the entire dataset. At this point, the interviews were read again to ensure that the content of the interviews is reflected by the themes and to include any uncoded passages. In a fifth step, we finalized the title of each theme and subtheme and defined each theme with clear and precise sentences. Several quotations from the interviewees were chosen as examples for each theme and to support the interpretation to our findings (sixth step). We discussed the theme building process at each step within our research group and repeatedly revisited the transcripts and developing themes. This iterative and reflexive approach allowed us to refine our thematic structure and ensure that it remained well supported by the data.

## Results

A total of 22 interviews were conducted (n = 11 with GPs, n = 6 with cardiologists, n = 5 with neurologists; one GP withdrew) (Table 1). Interviews were carried out from September 2023 through March 2024 and lasted between 19 and 53 minutes. Interviews were either held via video call (n = 16) or in person (n = 6), depending on the preference of the interviewees and the local distance between the interview partners. Physicians were between 37 and 68 years old and presented with a wide range of vocational experience (between 7 and 40 years). No small practices were included in our sample, as small GP practices seemed to become less common in recent years. Individual demographic details can be found in S3 Table.

**Table 1:** Characteristics of interviewed physicians.

		N = 22
Gender	Women	5
	Men	17
Age in years (median)		37–68 (56.5)
Region	Middle-, North-Hesse	12
	Northern Bavaria	10
Specialty	GPs	11
	Cardiologists	6
	Neurologists	5
Practical experience in years (median)		7-40 (26)
Type of practice	Single	10
	Joint	12
Practice size*	Small	–
	Medium	8
	Large	14
Setting	Urban	13
	Rural	9

* Practice size is determined by health insurance certificates per quarter; small: < 900 certificates, medium: 900–1500 certificates, large: > 1500 certificates.

**Fig 1:**
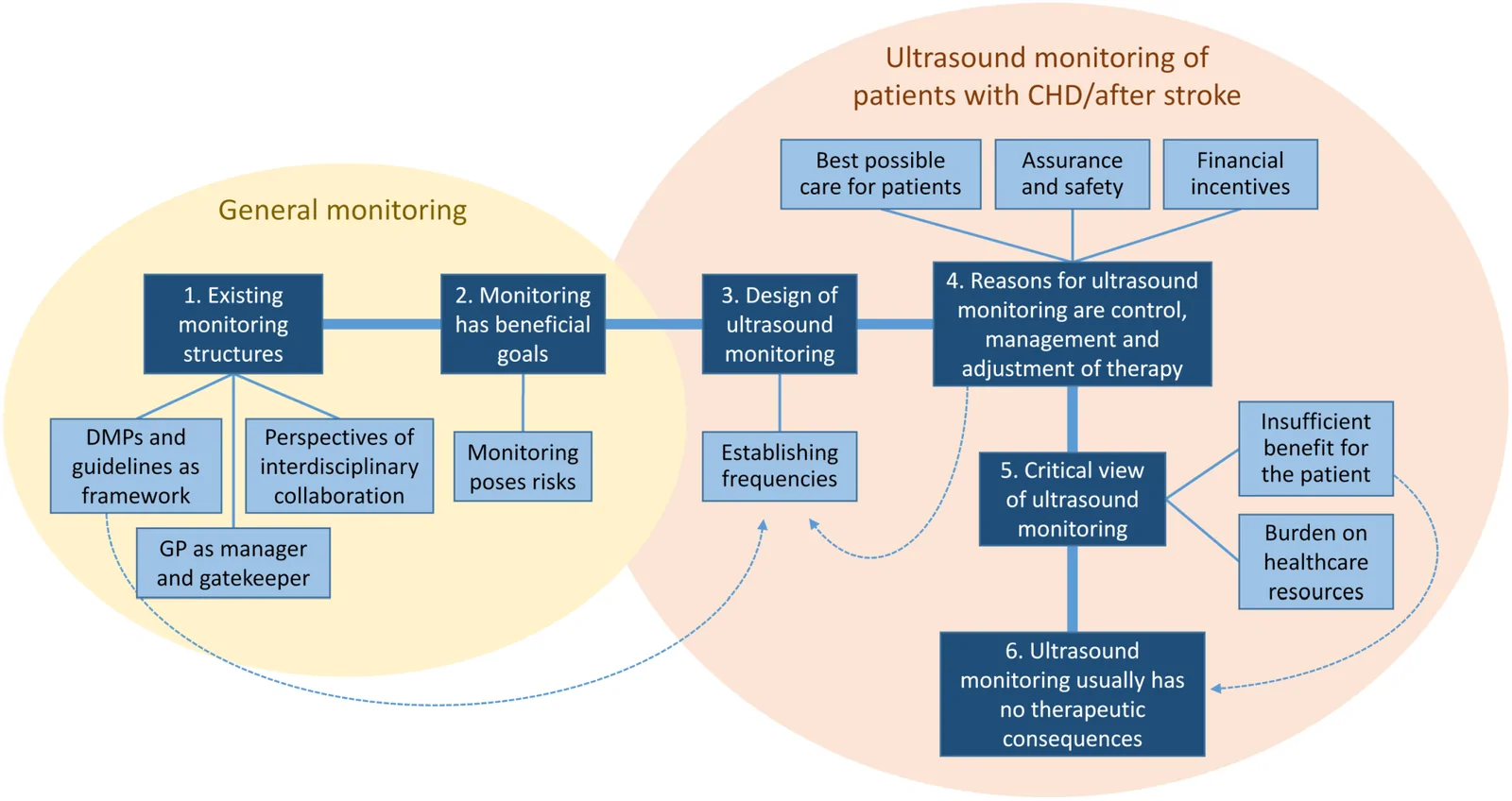
Themes and subthemes. Themes in dark blue, subthemes in light blue.

We identified six themes and up to three subthemes that were created from all interviews (Fig 1). Two themes addressed monitoring in general and reflect the structure and perspectives of monitoring procedures in the German healthcare system. Four themes were created with special focus on ultrasound monitoring of patients with CHD and after stroke, which portray the ambivalent view, motivations and consequences for these examinations.

### Theme 1: Existing monitoring structures

Monitoring in Germany requires collaboration between different specialised practices, e.g., general practices and independent outpatient cardiologists for additional examinations. Physicians from both institutions reported that they actively shared the care of the patient.

*“Patients come to the practice and often bring previous findings from their GP. And we/ If there are* *no previous findings from the GP, then we ask. If the patient says it hasn’t been done for a long* *time, hasn’t been checked, then we take care of it after consulting the GP, the check-ups, including cholesterol check-ups or blood pressure check-ups.” (C-15)*

Only a minority of patients were referred to specialized practices for monitoring examinations and received care at their GP practice.

*“So if I compare how frequent strokes are in the clinic, only a fraction of patients are seen by the neurologist. I would even say, just based on a feeling, maybe 5 to 10 percent of the patient* *population that’s actually out there.” (N-19)*

In addition to a range of regulations, as DMPs and guidelines, monitoring was also determined by the structure of the German healthcare system.

#### Subtheme: DMPs and guidelines as framework.

The design of monitoring routines could be determined by DMPs, guidelines or to some extent by the physicians themselves. Depending on the physicians’ specialty, this framework was either readily accepted, met with critical reflection or even rejection. GPs adopted DMPs and appreciated their advantages.

*“Well, I would definitely want to enrol him [the patient] in a DMP program because then I have my* *quarterly lab controls, my blood pressure control.” (GP-11)*

Most GPs stated that they were not following guidelines in general and that they were not firm in their content. Some GPs talked openly about not reading them, others felt caught out by not knowing their guidelines. One GP felt that more should be done than simply following guidelines, observing that some may not fully reflect the realities of everyday clinical practice.

*“There are guidelines. But I’m not a big fan of them. And I’m especially not a fan of having* *guidelines on a shelf somewhere. Or memorizing guidelines, so that’s not really my thing.” (GP-03)*

*“I’m not so guideline-oriented right now, eh?”* *(GP-09)*

*“And certain guidelines are just the same, they may be well designed, but they are out of touch with the world, so to speak, right? That’s why it’s called a guideline, it’s not an obligation, but* *sometimes you have to be aware that you’re breaching them.” (GP-02)*

Contrary to GPs, cardiologists mostly worked according to guidelines and were familiar with the details or aware of the lack of clear recommendations. They also were aware that guidelines have disadvantages, e.g., conflicts of interests or divergent statements between medical societies. Neurologists found guidelines in general important but said that they lack practicability and relevance specific for stroke patients. Some neurologists were therefore unsure about what they recommend regarding monitoring due to limited familiarity with the guidelines.

*“You’re grateful that you have them [guidelines]. I would do a damn thing not to follow the* *guidelines. But more guidelines will not shed more light, especially if they are not free of conflicts of interest.” (C-16)*

*“Guidelines? I’d have to think about it, yeah, how they write it there. But I can’t recall anything* *being specified, at least for stroke, about how often it should be checked each year.” (N-22)*

#### Subtheme: GPs as manager and gatekeeper.

Physicians in this study saw GPs as the first point of contact and coordinator in the care of patients and perceived that as positive.

*“As a general practitioner, I would say that all diseases should generally be monitored in the* *general practice.” (GP-09)*

*“So if the GP has a look, that’s a good thing. And then he can manage it a bit, whether he needs to* *see an internist or a diabetologist in particular or a cardiologist.” (N-22)*

#### Subtheme: Different perspectives and perceptions of interdisciplinary collaboration.

Collaboration between physicians of different specialties was characterized by mutual recognition. When asked about the quality of their collaboration, some GPs showed conditional respect, depending on earlier working relationships or clinical decisions.

*“Good with the people I work with. (laughs) So I basically picked a doctor in the background for* *every problem.” (GP-02)*

*“We are certainly open to suggestions from colleagues. And if we recognize that they make sense* *and that the patient’s compliance is there, we generally adopt them. Yes.” (GP-08)*

Critical views came from all specialties interviewed. They reported a lack of coordination and of feelings of being left out of their patients’ care. Mostly cardiologists said that treatment goals differed in GP practices from their own and recommendations were being ignored.

*“The cardiologist often handles that himself. Yes, he sees him [the patient] and: ‘Please come back in February 2024.’ And I’m completely out of the picture.” (GP-05)*

*“Unfortunately, we work surprisingly little with GPs. [...] The GPs also often have their own ideas, for example when it comes to cholesterol target values. There are divergent statements from the* *medical societies. And also regularly, yeah, the phenomenon that the medication instructions we give or the prescriptions we make are not continued.” (C-14)*

### Theme 2: Monitoring concepts has beneficial goals

In general, physicians viewed monitoring as beneficial for the patient and the health system. The goal of monitoring was seen as the early detection of deterioration in order to adjust therapy in a timely manner and thus delay progression of the chronic disease.

*“Yes, to recognize the progression of a chronic disease at an early stage. And to be able to intervene if the target values are not reached, to improve them with medication.” (GP-04)*

In the long term, physicians considered that monitored patients living with chronic conditions would be less likely to be hospitalised, thereby reducing healthcare costs.

*“So for the monitoring of cardiac insufficiency, heart failure, there is good data that patients don’t necessarily live longer, but that the number of hospital stays can be reduced. Which benefits everyone, yes, the patient, the healthcare system, the costs, everything, yes.” (C-14)*

#### Subtheme: Monitoring also poses risks.

When asked about negative aspects of monitoring, physicians suggested that too frequently performed monitoring can lead to overuse and to diagnostic cascades that can also be harmful for the patient.

*“The biggest risk of a monitoring examination. That a possibly over-interpreted finding leads to invasive measures being taken. Which would not necessarily have been done without this monitoring. That the patient’s health may even be put at risk.” (GP-03)*

*“The ultimate danger is overuse or overdiagnosis, which then quickly ends in overtreatment.” (N-21)*

### Theme 3: Design of ultrasound monitoring for patients with CHD/after stroke

Specialized physicians reported that they carry out the ultrasound monitoring. Some reported that there was no disease-specific monitoring concept specifically for the diseases investigated. Instead, physicians mainly relied on individual clinical judgement rather than standardized, condition-specific processes.

*“What if there was a process? Yes, wonderful, right? But I’m afraid that’s one of the areas where the/ let’s put it pathetically: the medical art still plays a role somehow.” (N-20)*

#### Subtheme: Establishing frequencies of ultrasound examinations.

Physicians reported that frequencies of ultrasound monitoring were determined by structural requirements (guidelines, DMPs or quartile billing system) as well as their own assessment and experience on the course of the disease or the patient’s wishes. Sometimes frequencies were established within a practice and were followed without further reflection. Younger physicians orientate themselves on their more experienced colleagues’ procedures. Physicians described that the monitoring frequency varied between three months and two years and was sometimes adjusted over the course of the disease.

*“As a cardiology practice, we would routinely see him once a year.” (C-13)*

*“On the one hand, how pronounced the symptoms are in that case. And on the other hand, how the course of the symptoms/ or the stenosis is. In other words, whether there is progression in the story.” (N-20)*

### Theme 4: Reasons for ultrasound monitoring are primarily control, management and adjustment of therapy

Reasons for ultrasound monitoring were diverse and individual, which led to the overarching theme of the main motivations for conducting these examinations. The factors primarily mentioned were progress control and management of the disease, as well as therapy adjustment if necessary. These factors also influenced the frequency of monitoring examinations.

*“The greatest benefit or the primary benefit from the understanding that I have is that I can adjust the treatment in the event of unexpected progression or previously unforeseeable problems or improvements.” (N-20)*

*“Yes, on the one hand an exclusion of deterioration or progression. That would be essential. Then to document for the future: Such and such were specific issues [...]. And then, of course, to keep reminding the patient: “You had something. You are a certain risk patient. Please pay attention to this and that so that it remains as stable as it is.” (C-14)*

#### Subtheme: Aiming for the best possible care for patients.

Physicians were aware of their patients’ wishes and addressed them with providing optimal care. They reported that most patients feel well cared for by close monitoring. Physicians therefore tended to enable their patients to undergo the examination.

*“The patient is closely monitored, they feel that they are in good hands and that their health is being watched. It is definitely very beneficial for their health if this is the case and if they are compliant and come to the practice.” (C-15)*

*“If it leads to a better feeling about life, because the cardiologist says again that everything is fine, and the system wants to afford it, then that’s the way it is.” (GP-02)*

#### Subtheme: Assurance and safety.

While the previous subtheme described physicians’ patient-oriented motivation for their care, this subtheme focuses on the need for assurance and safety, both for physicians and patients. On the one hand, with regular ultrasounds, physicians wanted to assure themselves that they did not miss anything. It seemed important that they were not overlooking relevant clinical changes or missing early signs of complications. Physicians felt mostly uncomfortable or considered it risky to omit ultrasound on symptom-free patients. It was also important to pick out a small group of patients who showed findings in the ultrasound.

*“And it’s kind of embarrassing when somehow wall movement vibrations or disorders are described to me a year later and you somehow don’t have an up-to-date echo.” (C-16)*

*“But most patients, they have unchanged echo findings when they come from last year to the next year, yes? So. Yes, but this small group of patients [in whom something changes] should also be fished out.” (C-17)*

*“I would see the [patients] every six months, in any case. It would be too precarious for me to see them only once a year.” (N-18)*

On the other hand, physicians were happy to accommodate their patients’ desire for safety. Physicians assumed that patients felt safer with regular ultrasounds and that this feeling was also beneficial to their health.

*“So, I also believe that the benefits are in the foreground and I believe that the patients cooperate better when they have close contact with the doctor. And they also feel safe and so it’s better to have too much than too little.” (C-17)*

*“I think that maybe one or two patients see it like a MOT certificate, right?” (GP-06)*

*“Somehow they feel more secure, yes, that yes, you’ve looked, yes, you’ve seen a picture, you’ve measured some parameters.” (N-22)*

#### Subtheme: Financial incentives driving monitoring routines.

Physicians considered the type of billing a significant contributor to the decision for an echocardiography or carotid ultrasound at a certain frequency. Financial incentives were seen as a key factor driving clinical practice and influencing monitoring routines. Cardiologist reported that, in Germany, the patient’s entire visit to the practice could be billed at a substantially higher rate if an ultrasound was included in the examination. Even when ultrasound was not included in a package, neurologists explained that it was carried out simply because it could be billed.

*“We do it [echocardiography], of course, because otherwise we wouldn’t get paid for the consultation here with the cardiologist. If we only do an ECG and a physical examination, we get a fraction of what we would get if we included ultrasound. Unfortunately, that’s the nature of the system.” (C-14)*

*“It’s just done because it’s billable. [...] I would probably also do it as a cardiologist, yes? No, it’s not a criticism in principle. People just do what makes them money.” (GP-05)*

*“So it’s clearly money-driven. So there are neurological practices that carry out ultrasound examinations even without a strict indication because they can be billed [...].” (N-21)*

### Theme 5: Critical view of ultrasound monitoring in CHD and stroke patients

Physicians from all specialties were critical of echocardiography or carotid ultrasound in the care of patients with CHD and after stroke.

*“There should be an indication to perform a carotid duplex. And not just like that, everyone has to have it at some point in their lives.” (GP-11)*

#### Subtheme: Insufficient benefit for the patient.

Despite the regular performance of ultrasound in uncomplicated CHD patients or in patients after a stroke due to carotid stenosis, several physicians saw no added value for this examination.

*“With heart failure, that makes sense. But in the case of pure CHD, if the patient has no other indications of cardiac insufficiency, it is completely meaningless. I don’t know, but it’s always done.” (GP-05)*

*“The most important thing is cholesterol management, blood pressure management, [...] I see relatively little added value here from a cardiologist, and I say that as a cardiologist. And many cardiologists say that behind closed doors. That’s just the way it is.” (C-16)*

*“So many people think that/ ultrasound is the main thing. But if the cause [for stroke] is not arteriosclerosis but cardiac arrhythmia, then that lulls some people into a false sense of security.” (N-18)*

#### Subtheme: Burden on resources in the healthcare system.

Physicians explained that performing ultrasounds on asymptomatic patients occupied their time and tied up resources, limiting their availability for acute patients and increasing costs in the healthcare system.

*“And if I have 20 returning patients a day who all have nothing and then I don’t have as many valences to care for patients with acute problems.” (C-12)*

*“The price for this, well, is a certain amount of effort. [...] A patient has to come to the practice. And a doctor, yes, has to take the time. Who would then also like to be compensated accordingly.” (N-20)*

### Theme 6: Ultrasound monitoring usually has no therapeutic consequences

Consequences of echocardiography in CHD patients without further morbidities were seen as rare. The measure was rather indicated for complications such as heart failure, wall motion disorders or valve defects. The consequences of duplex sonography were also considered to be very rare and only to be expected if the stroke was caused by a stenosis of the cervical vessels. Physicians thus perceived limited benefit for the patient. When directly asked about experienced consequences of echocardiography in CHD patients or carotid sonography in stroke patients, physicians generally denied it, emphasizing that such cases were extremely rare.

*“No, not in my case, because the cardiologists who are in the area [...] are very conservative.” (GP-02)*

*“I have to say quite honestly, generally, no, so not in the vast majority of cases.” (C-16)*

*“I’ve been in private practice for eight years now and it has happened to me exactly once.” (N-18)*

## Discussion

### Summary

In Germany, physicians from different specialties used different sources for guidance on monitoring patients with chronic conditions. GPs treated their patients along DMPs if available or based on their experience. In contrast, cardiologists and neurologists oriented their care procedures mostly based on guidelines supported by their clinical experience. GPs were seen as managers and gatekeepers in health care with patients being referred to other specialties from their GP. The German monitoring structure required collaboration across facility boundaries, which was characterized by pragmatic respect and understanding, but was also met with critical views regarding lack of coordination and differing treatment goals across specialties.

Monitoring was generally seen as beneficial for the patient and the health system. It was viewed as facilitating the early detection of deterioration, delaying disease progression through adaptation of therapy and leading to less hospitalisations. Physicians did bear in mind the risks of monitoring like overdiagnosis, overuse and overtreatment when examinations are done too frequently. Reasons and motivations for ultrasound monitoring in CHD and stroke patients were diverse and ranged from personal feelings and values, like aiming for the best possible care to own assurance and the patients’ safety to external factors like financial incentives. Despite this range of reasons, ultrasound as regular monitoring procedure was viewed controversially, as it showed insufficient medical benefits for the patient and it was a burden on resources like time and medical personnel. Echocardiography/carotid ultrasound rarely showed therapeutic consequences in everyday life of all interviewed physicians.

### Comparison with existing literature

Despite the fact that monitoring in general was seen advantageous by the interviewed physicians, the regular use of echocardiography/carotid ultrasound as part of monitoring routine was criticized, often being not indicated, and rarely resulted in further treatment consequences. Nevertheless, it was described by the participants as an established practice in the monitoring of non-symptomatic CHD and post-stroke patients.

Reasons that justified high levels of monitoring were diverse. In other studies, GPs reported fear and pressure of missing a disease as reasons for overtesting [27–30]. Additionally, GPs would often strive to fulfil patients’ demands for investigations [31], even when being aware that excessive testing can cause harm, e.g., worsen patients’ anxiety or lead to false positive findings [27,28]. The importance of routine examinations is reflected in the language used by both patients and physicians. In our parallel interview study of patients, participants compared monitoring with an annual motor vehicle check, a metaphor that also emerged in our physician interviews and has also been reported elsewhere [17,32]. Patients tend to overestimate intervention benefits, feel safer with more examinations and have trouble understanding overuse [17,33,34]. Furthermore, GPs report about pressure from other healthcare professionals to perform unnecessary tests on asymptomatic patients [27], a factor that we also observe in our interviews.

A major consequence of a high level of monitoring is an increased workload and strain on healthcare resources. While current guidelines provide numerous recommendations to intensify rather than de-intensify routine services [35], it is unrealistic to implement all these recommendations in practice due to time limitations [30,36]. Declining a patient’s request could have significant consequences and GPs report that some longstanding and close doctor-patient relationships have been harmed or even severed [32,37]. Physicians need effective strategies to communicate the necessity of fewer examinations to their patients [32].

The German clinical guideline for chronic CHD introduced a new chapter on monitoring in their 2024 update where no diagnostic intervention (including ergometry and echocardiography) was recommended in asymptomatic patients to assess stenosing CHD [11]. The fact that the echocardiography is part of a cardiology billing complex (that could only be billed as a package) is an example of a wasteful service: it does not deliver recognizable benefits and the incentive misaligns with health care system goals [38].

To address the issue of overdiagnosis, several strategies have been proposed. Increasing public awareness through targeted campaigns and promoting education on the benefits and harms of early diagnosis could help individuals make informed decisions [39]. Additionally, continuous medical education for both students and practitioners are essential to ensure a balanced approach to diagnosis and treatment. The evaluation of diagnostic technologies is crucial for the appropriate administration of increasingly sensitive tests [39]. Identifying targets for de-intensification and synthesizing evidence on unnecessary treatments should be incorporated into clinical guidelines [40]. These guidelines should provide clear recommendations to avoid or discontinue treatments when evidence for their benefit is weak, and potential harms are more likely [9,40]. Furthermore, the concept of “time needed to treat” [36] is often overlooked in guidelines, making the implementation of certain recommendations unrealistic [36]. Political action is also necessary to reform healthcare incentives, shifting the focus from quantity to quality of care [38,39]. Policymakers should foster an environment that prioritizes the provision of appropriate services rather than their sheer volume, for instance, by adopting payment models that promote value-based care across all stages of treatment [38,41].

### Strengths and limitations

An important strength of this study was that the interviews were held with physicians from different specializations (GPs, cardiologists, neurologists) who regularly performed monitoring routines to gain more insight into their beliefs and motivations, allowing us to capture perspectives beyond those of a single specialty. Interviewed physicians also had a wide age range and differed in their vocational experiences. Interviews were held in two federal states of Germany to ensure that procedures in the health care systems were not specific for a single state. Another strength was that the interview guide and the results of the analysis were discussed in a multi-professional team (GPs, psychologists and public health scientists that all had experience in qualitative research) not only within the project but also across two institutes of primary care. The self-reflection that the interviewees were willing to share with the interviewers about possibly unnecessary examinations in their own practices and their own personal beliefs and motivations, showed that the physicians did not want to please, but felt free and relaxed to talk. The non-medical background of interviewers probably led to physicians explaining more detailed, e.g., about the billing system and its influence on performing examinations. On the other hand, this could have had a negative effect: as the interviewers were not peers, some nuances of professional reasoning may not have been fully captured.

Further limitations had to be taken into account. Only physicians interested in research took part in the interview study which could have led to a selection bias. Certain character traits, such as being open to new things vs. traditional, or the placement of their doctor-patient relationship were not considered. It was possible that certain types of physicians were not represented and that their motivations for carrying out certain examinations would have offered alternative or differing points of view. Furthermore, physicians from other specialties also could have had additional perspectives. The results offer valuable insights for both clinical practice and health policy, and may help optimize monitoring strategies in CHD and stroke care.

## Conclusions

Ultrasound monitoring of CHD and stroke patients is performed due to physicians’ personal assurance, safety concerns for their patients and financial incentives. The existing billing system is a structural issue in the German healthcare system as it can influence physicians’ decisions independently of patient need. The medical benefits of ultrasounds appear limited for asymptomatic CHD and stroke patients. The routine use of echocardiography and carotid ultrasound rarely leads to therapeutic consequences, while resulting in a burden on resources in daily practice and the healthcare system. In order to achieve a more efficient and balanced use of monitoring examinations, systematic changes are required at a political level, particularly in the reimbursement structures. Rather than holding individual physicians accountable, the whole system needs to be reformed to match clinical practice to the evidence of examinations. Further research is needed to evaluate current monitoring routines and incorporate evidence-based recommendations into guidelines that optimize patient care and resource use.

## Supporting information

S1 Table COREQ checklist. (DOCX)

S2 Table Interview guide. (DOCX)

S3 Table Individual characteristics of interviewed physicians. (DOCX)

## Abbreviations

C: cardiologist
CHD: coronary heart disease
DMP: disease management program
GP: general practitioner
MOT: British annual vehicle inspection, equivalent to the German TÜV
N: neurologist
