# Supplementary material for: Motivations, experiences and perspectives of physicians in Germany regarding monitoring routines in coronary heart disease and post-stroke patients: A qualitative interview study
Source: PLoS One. 2026 Sep 11;21(9):e0348530. doi: 10.1371/journal.pone.0348530 (PMC13567794; doi:10.1371/journal.pone.0348530)
Supplement: Supplementary file 2 [file pone.0348530.s002.docx]

S2 Table. Interview guide.

**Initiation**

Introduction of interviewer and subject of this interview: monitoring patients with a chronic condition.

Statement that interviewer is not a physician and might ask more questions to understand medical proceedings.

**Main topic: Monitoring in general**

Questions: What do you define as monitoring?

What are typical diseases for monitoring?

What monitoring examinations do you do in your practice?

Are there advantages and disadvantages to monitoring?

**Main topic: Motivations for monitoring of patients with CHD/after stroke**

*Time to read example patient 1 (see table below).*

Questions: What examinations would this patient receive?

Do you have a standard procedure, anamnesis questions, examinations, frequency

Are there patients where you deviate from your standard procedure?

Are there guidelines or other regulations that you follow?

Does the monitoring take place in your practice or in the practices of other specialties?

Would there be any differences if the patient was older/had another event two years later/ a woman?

*Time to read example patient 2 (see table below).*

Would you proceed differently with this patient and why?

When we talk about patients in your practice, do you conduct any further examinations? How often?

What is the basis for your decisions?

How do you deal with uncertainties?

**Main topic: Collaboration with physicians of other specialties and patients**

Questions: How does the cooperation with specialists work?

Do you have the feeling that monitoring is bypassing you?

How do you rate the monitoring of the specialists/GPs?

Are there recommendations from specialists?

Do you implement the recommendations of specialists 100%?

How do you experience the cooperation with the patient?

Is monitoring usually initiated by you or is it actively requested by patients?

Are there differences according to patient type?

What do patients want from you?

**Main topic: Consequences of monitoring**

Questions: Do the monitoring measures have consequences for CHD/stroke patients?

Are there any consequences of echocardiography in CHD patients/carotid ultrasound in patients after stroke?

What exactly are the consequences of monitoring? Diagnostic? Therapeutic? Frequency?

In summary, how do you rate echocardiography/carotid ultrasound as a monitoring measure?

What goals do you think monitoring measures should have?

**End**

What is the biggest risk of monitoring for you? What is the greatest benefit?

- Specifically related to echocardiography/carotid ultrasound.
- Overall related to monitoring.

Is there anything else you would like to tell us?

Table: Fictional patient cases for interview focus on CHD and stroke

**Interview focus on CHD (GPs, cardiologists)**

**Fictional patient case 1 (CHD)**

Mr. P., 63 years old, store manager at Aldi, has been a regular visitor to your practice for many years. A glance at his list of diagnoses shows you Type 2 diabetes mellitus (non-insulin-dependent), arterial hypertension, hypercholesterolemia, post myocardial infarction 2015, benign prostatic hyperplasia, post knee replacement surgery. He regularly comes to your practice to pick up his prescriptions or visits you for acute problems such as sinusitis in the last quarter. He has been complaining of slight dizziness for years, but extensive investigations have not produced any clear results. Occasionally you ask about exertional dyspnea and angina pectoris, which he always denies. He lives with his wife and has a dog. You experience Mr. P. as friendly and open-minded and he gives the impression of being compliant.

**Fictional patient case 2 (CHD)**

Mr. A., 59 years old, has been a regular visitor to your practice for many years. His previous illness is arterial hypertension. During a manager check-up, he was diagnosed with high-grade coronary sclerosis, whereupon he received a stent. He always denies exertional dyspnea and angina pectoris. He is mobile without aids and lives alone in his apartment.

**Interview focus on stroke (GPs, neurologists)**

**Fictional patient case 1 (stroke)**

Mr. P., 67 years old, a vocational school teacher with early retirement has been one of your patients for many years. A glance at his list of diagnoses shows Type 2 diabetes mellitus (non-insulin-dependent), arterial hypertension, hypercholesterolemia, post ischemic stroke 2015 due to atrial fibrillation, hypothyroidism, post left knee replacement. He regularly comes to your practice to pick up his prescriptions or visits you for acute problems such as sinusitis in the last quarter. He has been complaining of slight dizziness for years, but extensive investigations have not produced any clear results. Hemiparesis of the upper right limb has largely recovered after the stroke. He lives with his wife and volunteers at events in the local community center. You experience Mr. P. as friendly and open-minded and he gives the impression of being compliant.

**Fictional patient case 2 (stroke)**

Mrs. A., 69 years old, has been coming to your practice regularly for many years. A glance at her list of diagnoses shows you arterial hypertension, mild aortic stenosis, right carotid stenosis 50%, heart failure NYHA II, post-apoplexy 2021. She is mobile without aids and lives alone in her apartment. Her daughter lives next door and helps her with cooking, shopping etc. She has had mild dysarthria since the stroke. She receives regular speech therapy for this.
