# Supplementary material for: Motivations, experiences and perspectives of physicians in Germany regarding monitoring routines in coronary heart disease and post-stroke patients: A qualitative interview study
Source: PLoS One. 2026 Sep 11;21(9):e0348530. doi: 10.1371/journal.pone.0348530 (PMC13567794; doi:10.1371/journal.pone.0348530)
Supplement: Supplementary file 3 [file pone.0348530.s003.docx]

S3 Table. Individual characteristics of interviewed physicians.

| **Physician** | **Specialty** | **Interview focus** | **Age (years)** | **Gender** | **Practical experience** | **Type of practice** | **Practice size**** | **Setting** |
| --- | --- | --- | --- | --- | --- | --- | --- | --- |
| GP-01 | GP | CHD | 63 | woman | 39 | single | large | urban |
| GP-02 | GP | CHD | 43 | man | 15 | joint | large | rural |
| GP-03 | GP | CHD | 63 | man | 36 | single | medium | rural |
| GP-04 | GP | CHD | 51 | man | 23 | single | medium | urban |
| GP-05 | GP | CHD | 44 | woman | 17 | single | medium | rural |
| GP-06 | GP | stroke | 58 | man | 32 | joint | large | rural |
| GP-07 | GP | stroke | 64 | man | 38 | joint | large | rural |
| GP-08 | GP | stroke | 64 | man | 35 | joint | large | urban |
| GP-09 | GP | stroke | 55 | man | 24 | joint | large | urban |
| GP-10 | GP | stroke | 68 | man | 40 | single | large | rural |
| GP-11 | GP | stroke | 62 | woman | 28 | single | large | rural |
| C-12 | Cardiologist | CHD | 63 | man | 37 | joint | large | urban |
| C-13 | Cardiologist | CHD | 68 | man | 37 | joint* | large | urban |
| C-14 | Cardiologist | CHD | 49 | man | 22 | joint | large | urban |
| C-15 | Cardiologist | CHD | 62 | man | 37 | single | medium | urban |
| C-16 | Cardiologist | CHD | 39 | man | 11 | single | large | rural |
| C-17 | Cardiologist | CHD | 37 | woman | 11 | single | medium | urban |
| N-18 | Neurologist | stroke | 45 | woman | 14 | single | medium | urban |
| N-19 | Neurologist | stroke | 37 | man | 7 | joint | large | rural |
| N-20 | Neurologist | stroke | 50 | man | 22 | joint | large | urban |
| N-21 | Neurologist | stroke | 58 | man | 29 | joint* | medium | urban |
| N-22 | Neurologist | stroke | 41 | man | 16 | joint | medium | urban |

GP: general practitioner; C: cardiologist; N: neurologist.

* MVZ (Medizinisches Versorgungszentrum): medical care center

** Practice size is determined by health insurance certificates per quarter; small: < 900 certificates, medium: 900-1500 certificates, large: > 1500 certificates.
