# Supplementary material for: Motivations, experiences and perspectives of physicians in Germany regarding monitoring routines in coronary heart disease and post-stroke patients: A qualitative interview study
Source: PLoS One. 2026 Sep 11;21(9):e0348530. doi: 10.1371/journal.pone.0348530 (PMC13567794; doi:10.1371/journal.pone.0348530)
Supplement: Supplementary file 4 [file pone.0348530.s004.docx]

**PONE-D-25-32189R2**

Monitoring routines for people with chronic conditions: Motivations, experiences and perspectives of physicians from different specialties - a qualitative interview study

**Revised title:**

Motivations, experiences and perspectives of physicians in Germany regarding monitoring routines in coronary heart disease and post-stroke patients: a qualitative interview study

Dear Reviewers,

thank you very much for the revision of our manuscript. We greatly appreciate the constructive feedback which helped us to improve the clarity and quality of our work. In the following, we have addressed each comment and suggestion in detail and made corresponding revisions to the manuscript. All line numbers refer to the manuscript with track changes.

Yours sincerely,

Julia Heisig

**Reviewer #3:**

The manuscript addresses a sensitive and clinically important issue from the perspectives of patients, clinicians, and the healthcare system. The study is methodologically sound: the design is appropriate to the research objectives, and the methods are clearly described and justified. The manuscript is well written and easy to follow, and the inclusion of direct quotations from interviewees enhances transparency and facilitates a deeper understanding of the findings.

The authors provide a thorough comparison of their results with the existing literature and present a balanced discussion of the study’s strengths and limitations. The Conclusions section appropriately highlights key questions emerging from the findings and underscores the need for further research, particularly to evaluate current monitoring practices and to integrate evidence-based recommendations into clinical guidelines aimed at optimizing patient outcomes and resource utilization.

To further strengthen the manuscript, it would be valuable for the authors to articulate more explicitly the novel contribution of this study to the existing body of knowledge. A clearer statement of what specifically advances current understanding would enhance the overall impact and relevance of the work.

We thank the reviewer for this important suggestion. We have now added a clearer statement in the Introduction to explicitly highlight the novel contribution of our study (lines 97-103).

Building on previous work showing that many monitoring routines in chronic conditions lack a strong evidence base, our study adds a multi-perspective explanation of why such routines are still performed in clinical practice. Specifically, within the ChroMo project we combine physicians’ interviews (this manuscript), patients’ perspectives, and complementary routine data to explore motivations and perceived benefits of monitoring routines across different specialties and two diseases.

This paper specifically contributes an in-depth understanding of physicians’ perspectives on why routine imaging monitoring may continue in clinical practice despite limited evidence. By including GPs, cardiologists and neurologists, and by focusing on two clinical examples, coronary heart disease and post-stroke care, the study highlights how clinical reasoning, perceived patient needs, safety concerns, collaboration patterns and reimbursement structures interact in everyday monitoring practices.

**Reviewer #4:**

Monitoring routines for people with chronic conditions: Motivations, experiences and perspectives of physicians from different specialties - a qualitative interview study

By Heisig et. al.

The paper concerns interviews over the relevance and timeliness of monitoring routines for chronic heart disease (CHD) and stroke. My immediate impression is that the paper is relevant, with the survey appropriately carried out, and, to some extent, appropriately summarized. There are however in my opinion a number of things that the authors should address prior to publication. In particular the discussion section appears weak, and would benefit from describing the results and their implications in light of existing literature, rather than the so-called 'comparison with existing literature' that is currently the main section of the discussion. Furthermore it appears to me that some of the conclusions are too strict from the presented evidence. Specific things to attend are listed below.

A. The title. The title indicate general monitoring routines for 'chronic conditions'. But this is not what the authors investigate. 'Chronic diseases' are limited to two diseases, CHD and stroke, which are both diseases from the circulary system, and the authors have not justified nor discussed overlap with general chronic diseases in the monitoring routines. This should be reflected in the title.

Thank you for your feedback. We agree with your reasoning and changed the title accordingly. We removed the expression “chronic conditions” and stated the two diseases we investigated.

Revised full title:

Motivations, experiences and perspectives of physicians in Germany regarding monitoring routines in coronary heart disease and post-stroke patients: a qualitative interview study

Revised short title:

Physicians’ views on monitoring routines in coronary heart disease and post-stroke patients

B. How have cardiologists and neurologists been selected? The text only describe how they are contacted (email found through web page), but not why the individual cariologists/neurologists were contacted.

We added more details to our recruitment strategy to make it clearer how cardiologists and neurologists were recruited. We added the following paragraph (line 126):

Cardiologists and neurologists from the local region were initially approached; if no response was received within a few days, recruitment was expanded to a broader geographic area within Northern- and Middle-Hesse and Northern Bavaria. Recruitment efforts were finalised when all participants appeared well-balanced regarding practice setting (region, rural/urban and single/joint), specialty, age and practical experience.

C. The structure of the 'Data analysis' section should be tightened. The authors decide to describe 6 steps in a reference to a thematic approach to qualitative analysis. Steps 1-3 and 5 are numbered in the description, but step 4 is referred to as 'the next step' and the contents of step 6 is not specified. Please tighten up this notation.

We added the numeration of each step in lines 190 and 195.

D. The interviews are grouped to GPs, cardiologists and neurologists. However, when going over the results, it appears that in particular neurologists are not mentioned. For example, in the 'Subtheme: DMPs and guidelines as framework', the GP attitude and cardiologist attitudes are scrutinized, but neurologists are not mentioned. Despite what seems to be a quote from a neurologist (N-19). Attitudes should be described either as a general trend with specification of deviations among the groups, or be mentioning all three groups. Otherwise the results are partial.

We added a paragraph to describe the neurologists‘ view (line 255). Neurologists found guidelines in general important, but guidelines either lack specific working instructions or neurologist are unsure what they say due to not reading them for a long time.

In the following themes we did not specify between cardiologists and neurologists, whenever their views overlap and could be combined. There was no benefit to distinguish between these two specialties and we refer to both as “physicians” (often together with GPs).

The paragraph in line 253 made sense to add as there was a difference between cardiologists and neurologists in how they use guidelines. Thank you for pointing this out.

E. In light of the results in Theme 4, I will question whether the authors have evidence for writing that the regular use of EKG/ultrasound was considered unnecessary. Theme 4 lists benefits from monitoring with EKC/ultrasound, and while other themes may indicate attitudes that support that such monitoring is unnecessary, this may be voiced at other interviews than those that lists benefits in Theme 4, essentially dividing the sample population into two heterogeneous groups, where one group voice benefits and the other that it is unnecessary. The authors should argue from their thematic approach that this is not the case, if the evaluation of the EKG/ultrasound as unnecessary is maintained.

We agree with the reviewer that the term “unnecessary” does not adequately reflect the complexity of the findings. The interviewed physicians said that monitoring of asymptomatic CHD and post-stroke patients has benefits but these benefits are not directly connected to medical consequences of these examinations. The benefits are overarching like meeting their patients need for safety or their own need for assurance to not miss anything. An additional driving force for monitoring routines are the financial incentives. The medical necessity of these examinations is subordinated. But we also agree that “unnecessary” does not fit and should be avoided as it does not reflect the complexity of what are the driving forces behind monitoring routines.

We adjusted the wording in line 454 and 507 to avoid or weaken the expression “unnecessary”. We agree that it now better reflects the physicians’ views.

F. The use of past tense in the section 'comparison with existing literature'. Here it is difficult to distinguish when the authors refer to results from the literature, and when it is aspects of their own results that is discussed. In terms of what the comparison means prospectively, it is odd that past tense is used. For example lines 464-467, where it is (was) essential with continuous medical education and evaluation of diagnostic technologies.

The whole discussion section was revised and the use of simple present and past tense was corrected.

G. I am curious as to why patients perception of intervention benefits is discussed in lines 445-448 as it is. There are no patients in this study, only the thoughts from the physicians about such, which may be a very different thing. The discussion should refflect that.

We agree that the present study reports physicians’ perspectives and does not directly investigate patients’ views. However, we feel it is important to emphasize that the physicians’ views about their patients were also seen in the patients’ perspective. Especially, as both perspectives are investigated in the ChroMo study: the physicians’ and patients’ interview study are both sub projects of the ChroMo study. The recited paper [17] refers to the interview study of patients, conducted by our group at the same time. We added more explanation as to why we included the patients’ views in the discussion (line 461). The following sentence describes the reasons why monitoring is important for patients that was also described in our patients’ article and other studies.

Note: the reference given in brackets (Rink et al. 2025, under review) was removed and incorporated as a citation [17], as the article has been recently published.

H. I believe that the authors in a number of passages are overstating the evidence from the survey. In line 449, they mention 'overtesting'; but do their data data support that overtesting takes place, whether overtesting is the rule? And what do the authors consider as 'overtesting'? Needless to say, more testing than needed, but when is the benefit sufficiently small to rate a test procedure as 'overtesting'?

Thank you for pointing this out. We appreciate your concern regarding the use of the term "overtesting." Some interviewed physicians used this term directly (not cited), and we believe the use of this word reflects the perspective from our interviews.

We agree that we should avoid overstating our findings. We used the term only in the discussion section when reflecting our results in the context of other articles that investigated overtesting, as their findings showed parallels to our results.

We revised the wording (lines 457, 469) and now used the term only when reflecting the results of other studies.

I. In the S&L section, the authors claim that the work captures 'perspectives that extend beyond the viewpoint of any single speciality'. Considering that the authors only look into 2 specialities, which are even for the same group of diseases, I think that such a claim is stretching it a bit, cf. my view on the title of the paper. The authors should argue for that the information gathered can be formulated in such a broad way. It is worth noting that the authors inline 499 state that 'physicians from other specialities also could have additional perspectives'. Yes indeed.

We agree that the original wording may have overstated the breadth of perspectives captured in our study. Therefore, we revised the sentence to a more cautious formulation and now state that the study captured perspectives beyond those of a single specialty (line 501).

We would also like to clarify that the study included physicians from three specialties: general medicine, cardiology, and neurology. In Germany, general medicine is a medical specialty with a structured vocational training program comparable in duration and organization to those of cardiology and neurology. Nevertheless, we agree that physicians from other specialties may have additional perspectives, as discussed in the limitations section.

J. In the conclusion I believe that the authors overstate the evidence from the survey. For example, they write that 'The medical benefits of ultrasound are limited for CHD and stroke patients'. The authors cannot know that. The evidence is at the most that the benefit APPEARS limited from the survey, but to draw a conclusion beyond the material, and indeed over all interviewees, as as I see it unfounded.

We agree that this sentence overstated our findings. We therefore revised the wording to weaken the statement and specified that it refers to asymptomatic patients. The sentence now reads:

The medical benefits of ultrasounds appear limited for asymptomatic CHD and stroke patients (line 526).

K. In the abstract, the authors state that 'transcripts were analysed with thematic analysis according to Braun and Clarke'. 'Braun and Clarke' is here the authors of a publication that the authors refer to wrt. thematic analysis. However, in the abstract, these names should not appear, thematic analysis in itself should be enough, as the reference may be found in the main text. Laypersons may be mislead to think that 'Braun and Clarke' is a principle rather than a reference, which there by the way is two of (two papers of Braun and Clarke).

We removed the authors “Braun and Clarke” as we agree that it is not necessary to mention authors in an abstract (line 32).

L. Grammar: Line 47, line 130.

Thank you for your helpful input, we corrected both errors accordingly (now line 51 and 145).
