# Supplementary material for: Motivations, experiences and perspectives of physicians in Germany regarding monitoring routines in coronary heart disease and post-stroke patients: A qualitative interview study
Source: PLoS One. 2026 Sep 11;21(9):e0348530. doi: 10.1371/journal.pone.0348530 (PMC13567794; doi:10.1371/journal.pone.0348530)
Supplement: Peer review history

# Peer review: Decision Letter 0

**DOI:** 10.1371/journal.pone.0348530.r001

**Authors:** Ahmed Qasim Mohammed Alhatemi

14 Sep 2025

Dear Dr. Heisig,

Thank you for submitting your manuscript to PLOS ONE. After careful consideration, we feel that it has merit but does not fully meet PLOS ONE’s publication criteria as it currently stands. Therefore, we invite you to submit a revised version of the manuscript that addresses the points raised during the review process.

plosone@plos.org. When you're ready to submit your revision, log on to https://www.editorialmanager.com/pone/ and select the 'Submissions Needing Revision' folder to locate your manuscript file.

- A rebuttal letter that responds to each point raised by the academic editor and reviewer(s). You should upload this letter as a separate file labeled 'Response to Reviewers'.
- A marked-up copy of your manuscript that highlights changes made to the original version. You should upload this as a separate file labeled 'Revised Manuscript with Track Changes'.
- An unmarked version of your revised paper without tracked changes. You should upload this as a separate file labeled 'Manuscript'.

If applicable, we recommend that you deposit your laboratory protocols in protocols.io to enhance the reproducibility of your results. Protocols.io assigns your protocol its own identifier (DOI) so that it can be cited independently in the future. For instructions see: https://journals.plos.org/plosone/s/submission-guidelines#loc-laboratory-protocols. Additionally, PLOS ONE offers an option for publishing peer-reviewed Lab Protocol articles, which describe protocols hosted on protocols.io. Read more information on sharing protocols at https://plos.org/protocols?utm_medium=editorial-email&utm_source=authorletters&utm_campaign=protocols.

We look forward to receiving your revised manuscript.

Kind regards,

Ahmed Qasim Mohammed Alhatemi, Mbchb

Academic Editor

PLOS ONE

Journal Requirements:

When submitting your revision, we need you to address these additional requirements.

1. Please ensure that your manuscript meets PLOS ONE's style requirements, including those for file naming. The PLOS ONE style templates can be found at

https://journals.plos.org/plosone/s/file?id=wjVg/PLOSOne_formatting_sample_main_body.pdf and

https://journals.plos.org/plosone/s/file?id=ba62/PLOSOne_formatting_sample_title_authors_affiliations.pdf

2. We note that the grant information you provided in the ‘Funding Information’ and ‘Financial Disclosure’ sections do not match.

When you resubmit, please ensure that you provide the correct grant numbers for the awards you received for your study in the ‘Funding Information’ section.

3. Thank you for stating the following in the Acknowledgments Section of your manuscript:

The authors would like to thank all physicians for participation in this interview study. The authors would also like to thank all researchers from the Institutes who participated in the analysis sessions. We acknowledge financial support by “Deutsche Forschungsgemeinschaft (DFG)” and University of Marburg within the funding programme “Open Access Publication Funding”.

We note that you have provided funding information that is not currently declared in your Funding Statement. However, funding information should not appear in the Acknowledgments section or other areas of your manuscript. We will only publish funding information present in the Funding Statement section of the online submission form.

Please remove any funding-related text from the manuscript and let us know how you would like to update your Funding Statement. Currently, your Funding Statement reads as follows:

The research presented in this article was part of the project ChroMo (Monitoring routines for people with chronic diseases: Assessment and roadmap for the future) and was funded by the German Innovation Fund of the Federal Joint Committee (G-BA) [grant number 01VSF22046]. The funders had no role in study design, data collection and analysis, decision to publish, or preparation of the manuscript.

Please include your amended statements within your cover letter; we will change the online submission form on your behalf.

4. In this instance it seems there may be acceptable restrictions in place that prevent the public sharing of your minimal data. However, in line with our goal of ensuring long-term data availability to all interested researchers, PLOS’ Data Policy states that authors cannot be the sole named individuals responsible for ensuring data access (http://journals.plos.org/plosone/s/data-availability#loc-acceptable-data-sharing-methods).

Data requests to a non-author institutional point of contact, such as a data access or ethics committee, helps guarantee long term stability and availability of data. Providing interested researchers with a durable point of contact ensures data will be accessible even if an author changes email addresses, institutions, or becomes unavailable to answer requests.

Before we proceed with your manuscript, please also provide non-author contact information (phone/email/hyperlink) for a data access committee, ethics committee, or other institutional body to which data requests may be sent. If no institutional body is available to respond to requests for your minimal data, please consider if there any institutional representatives who did not collaborate in the study, and are not listed as authors on the manuscript, who would be able to hold the data and respond to external requests for data access? If so, please provide their contact information (i.e., email address). Please also provide details on how you will ensure persistent or long-term data storage and availability.

5. Please include captions for your Supporting Information files at the end of your manuscript, and update any in-text citations to match accordingly. Please see our Supporting Information guidelines for more information: http://journals.plos.org/plosone/s/supporting-information.

6. Please remove all personal information, ensure that the data shared are in accordance with participant consent, and re-upload a fully anonymized data set.

Note: spreadsheet columns with personal information must be removed and not hidden as all hidden columns will appear in the published file.

Additional guidance on preparing raw data for publication can be found in our Data Policy (https://journals.plos.org/plosone/s/data-availability#loc-human-research-participant-data-and-other-sensitive-data) and in the following article: http://www.bmj.com/content/340/bmj.c181.long.

7. If the reviewer comments include a recommendation to cite specific previously published works, please review and evaluate these publications to determine whether they are relevant and should be cited. There is no requirement to cite these works unless the editor has indicated otherwise.

8. Please review your reference list to ensure that it is complete and correct. If you have cited papers that have been retracted, please include the rationale for doing so in the manuscript text, or remove these references and replace them with relevant current references. Any changes to the reference list should be mentioned in the rebuttal letter that accompanies your revised manuscript. If you need to cite a retracted article, indicate the article’s retracted status in the References list and also include a citation and full reference for the retraction notice.

[Note: HTML markup is below. Please do not edit.]

Reviewers' comments:

Reviewer's Responses to Questions

**Comments to the Author**

1. Is the manuscript technically sound, and do the data support the conclusions?

Reviewer #1: Yes

Reviewer #2: Yes

**********

2. Has the statistical analysis been performed appropriately and rigorously?

Reviewer #1: N/A

Reviewer #2: N/A

**********

3. Have the authors made all data underlying the findings in their manuscript fully available?

The PLOS Data policy

Reviewer #1: Yes

Reviewer #2: No

**********

4. Is the manuscript presented in an intelligible fashion and written in standard English?

Reviewer #1: Yes

Reviewer #2: Yes

**********

Reviewer #1: General comments: interesting manuscript well written but elements missing about the design which decreases the rigor of the presentation. The data analysis analysis is appropriate.

Line 27: the authors should add more information about the design which will be used not only the method of data collection

Line 49: add information why you put cancer (reference, some are in remission and the monitoring is different based on the type of cancer, etc.)

Great details about the German health care system (lines 56-68)

Lines 79-86: provide the rationale to chose these 2 conditions; is it related to their incidence in your country or....

Line 93: requirement to add information about the type of design leading to the semi-structured interview; more details required about the qualitative design

Line 112: sample size? any details about your target number, references to support whatever you are coming up with

Line 114: what did you use to develop your interview guide? frameworks, theory or only experiences?

Line 134: did you use data saturation principle or clinical saturation; important to guide it with evidence

Line 158: did you ask the participants to review their transcripts? if not why?

Line 171: how many subthemes and why so many?

Line 227 and other: when you start a new subtheme can you indicate in your summary which type (s) of physicians your are focusing on because sometimes the quotes are from the GPs, cardiologists or neurologists, randomly

and your summary always start with physicians

some misleading information for the reader as each physician provides a different perspective; so make sure to link the quotes with the description

Line 275 and the figure can you highlight the link with the DMP and the figure because of the arrows

Line 301: can you explain what you mean by assurance in more details as you did for safety after

GREAT summary at the end and I like the way the authors compare with existing literature

Line 438 can you expand of what you mean about the following: "independent on their specialty"

Reviewer #2: Thank you for the opportunity to read your paper. It’s a really interesting piece of work and it is well written and structured, and for the most part, is presented clearly. I believe this fits well with Plos One, addresses the Criteria for Publication and would make interesting reading for the Plos One readership and more broadly, those interested in this field.

I've suggested the minor revisions below (including comments / questions), structured under the headings of each section of your paper. All are offered with the intention to enhance and provide additional clarity on the work you have done. The main area where further content / greater clarity on your work is needed is the methods and results.

Title & Abstract

Title

Perhaps add ‘influences’ into the title given the focus in the paper on external influences (policy, funding / reimbursement) on physicians motivations, actions etc.

Abstract

22,23 – to support clarity and flow change to “which negatively impacts upon patients quality of life and increases burden on healthcare systems” or something similar.

25 – remove ‘and ‘ so sentence reads “…benefit for the patient, detect deterioration and guide treatment adaptations”

31 – add how the interview data was analysed, add reference to following COREQ and Stenfors (2020) reference.

29 – change viewed controversially to “considered as controversial”

43, 44 – I think it’s unclear what reimbursement structures means in the abstract, while its explained in the main body of the text, for the abstract I think this could be reworded or briefly explained for clarity.

44 – not sure what “complex” means here, is it that this is about technically complex monitoring routines and / or is it the context and individual interpretations / applications that are complex?

Introduction

General point – consistency between use of this study, this interview study etc. perhaps for clarity refer to this paper and its focus. For example, “this paper focuses on interviews with physicians, we have published a separate paper from the ChroMO study which presents patients views etc…”

49, 50 – perhaps reword to “…place a burden on both patients’ well-being and quality of life as well as the healthcare system” or as suggested above.

76 – is there a reference that could be added here to support your point?

77, 78 – provide full name of the study before abbreviation, perhaps add some further information on what specifically ChroMO involves, other methods etc. or include link to website / study page / other publications will support the reader to gain a more detailed understanding of the wider work or add reference which provides this information.

83, 84 – add reference regarding your point on there being no clear benefit and negative impact on patients.

85, 86 – add reference to your other work regarding patients perspectives.

77, 86 – rewording is needed here to clarify the different projects and what they specifically focus on, It’s important to make it as clear as possible that the patient focused work is the focus of your other paper.

87, 91 – was there only one RQ? Were there any secondary Q’s? Could this one question be broken so that it aligns with the structure of the topic guide?

For example:

1. What are physicians motivations for monitoring of patients with CHD/after stroke?

2. How do physicians collaborate with other physicians and with patients?

3. What are consequences of monitoring (including benefits and risks)?

Methods

Study Design

94 – change wording to “we conducted semi structured interviews with GPs”

96-97 – to better align with Plos Ones criteria for publication regarding clarity of methods and reproducibility more is needed here regarding how Stenfors (2020) position on qualitative research has informed your work as well as its compatibility with Braun and Clarke’s TA, see Toward good practice in thematic analysis: Avoiding common problems and be(com)ing a knowing researcher, Braun and Clarke 2022.

Study Sample

General – how were physicians identified / chosen by the study centres, how were physician emails accessed? How many physicians were invited in total? How many declined, didn’t respond, accept but then not able to take part?

104 – change wording to ‘ The recruitment period for this study was…’

105 – change wording to “invitations were sent out via email through the research practice network….”

Data Collection

General – check tense through, focus on works that has already been complete.

116, 117 – clarify who the interview guide pretested with.

120 – add a brief explanation of which physicians completed which fictional patient case(s).

119, 122 – perhaps additional RQs can be derived here

123, 124 – clarify what video call software was used and reference if applicable.

132-134 – reference data saturation here although there are wider questions regarding whether data saturation (more of a positivist stance) aligns with Braun and Clarkes reflexive TA.

135 – who completed the transcription?

137 – include demographic survey as supplementary material.

142 – Braun and Clarke’s work from 2006 has since been updated, was this consulted?

144 – add reference to B&Cs work again.

147 – clarify who these institutes were.

150 – reword to ensure consistency with Braun and Clarke’s approach, rather than themes emerged, state that authors / researchers created themes.

156, 157 – transparency and trustworthiness, this perhaps does not related to Braun and Clarke’s approach instead reflecting a more positivist paradigm perhaps reword to support the interpretation of data via multiple viewpoints or something along these lines.

Results

General points

� Clear thematic structure throughout, quotes primarily support points made in the main body of the text (a couple noted below, could be removed / changed).

� Any way to draw out any difference in views between different physicians within / between groups?

167 – any reason for no small practices? Important to acknowledge and explain, briefly.

171 – Reword ‘emerged’ to created or designated etc. by the research team.

173 – reword emerged to created by the research team.

191-193 – repetition of point on cooperation across systems – perhaps reference guideline.

196, 197– edit sentence to support flow, as follows: “Depending on the physicians' specialty, this framework was either readily accepted, met with critical reflection or even rejection”

201, 210 – to support clarity for the reader my preference would be to separate out quotes and align with points in main point of text i.e. one quote per piece of text but I know Plos One do not follow this.

203 – add more nuance here RE quote, the out of touch point seems really important to emphasise.

211-212 – not sure what “…considered themselves firm in details” means, I think rewording to clarify your point here would be helpful for the reader.

220 – instead of “the medical community” say “physicians in this study”. The medical community implies a much larger population than included in this work.

250 – add additional point here about reduced burden on the healthcare system as a whole, was there anything here about workload burden on physicians too and the potential positive impact of monitoring in reducing it?

254-262 – can a point be added here which is the opposite of the point above, namely that too frequent monitoring creates additional system, staff burden etc.?

265-268 – change “…some told…” to “some reported that there were…” . Also, I’m not clear on how the quote reflects the point made before it, is there another quote that could be used to illustrate this point more clearly?

270-272 – reword for clarity here i.e. “Physicians reported that frequencies of ultrasound monitoring were determined by structural requirements (guidelines or quartile billing system) as well as their own assessment and experience on the course of the disease or the patient's wishes.”

279 – not sure that quote C-13 is adding much here, perhaps use other that outlines the variation in monitoring frequency.

282 – add additional words to the second sentence to aid flow and clarity i.e. “The factors primarily mentioned were progress control…” this is a really interesting point and would add analytical value if explored more, were their differences between / within physicians?

301 – remove contraction ‘don’t’ change to do not

312-320 – repeating the same point made in lines 292-299, could be integrated above and therefore cut down.

321 – I wonder whether a bit more explanation could be added to the sub-theme financial incentives, for example, ‘ Financial incentives driving clinical practice’

352 – perhaps sub-theme could be reworded to emphasise the wide impact across the health system for example “burden on the healthcare system”

372-73 – This quote from (N-18) does not deny the point made therefore I think this quote should be separated from the other two and further text added highlighting that not all physicians denied it.

Discussion

Summary

General point– review tense throughout to ensure consistency.

382 – clarify what you mean by “critical approach” as I do not think it is clear at the moment.

377-379 – slight rewording would help clarity here regarding difference between GPs and Cardiologists and Neurologists, for example: “GPs treated their patients along DMPs if they were available, however, they primarily treated patient based on their individual experience. In contrast, cardiologists and neurologists oriented their care procedures mostly based on guidelines supported by their clinical experience”

379-380 – slight rewording here for clarity would be helpful for the reader: “GPs were seen as the managers and gatekeepers in health care with patients being referred to other specialties by their GP.”

383-385 - slight rewording here for clarity would be helpful for the reader: “Monitoring was generally seen as beneficial for the patient and the health system. It was viewed as facilitating the early detection of deterioration, delaying disease progression through adaptation of therapy and leading to less hospitalizations.”

Comparison with existing literature

General point– any useful points to make regarding difference views between professions and alignment or difference with wider literature?

Rink et al (2025) reference to be updated if possible following outcome of review.

394-397- is this reported in the wider literature?

408 – A really important point RE increased workload, I think this should be highlighted further with additional quote(s) in the Results.

411-414 – Another important point regarding the impact of declining patients request, could any quotes / content be added to the results?

417-425 – I think its really important to bring in one of your important findings here regarding physicians openness about conducting an ultrasound because of the financial incentives. For example, this implies that the education of students and practitioners / physicians isn’t the issue as they are aware of its risks / lack of need at times. While this is a systemic issue, there’s also a behavioural / individual level challenge here.

429 – in-text reference should read (9, 32)

430 - “time needed to treat” is this a quote from an interview / paper? If so should be referenced / an ID provided.

432 – in-text reference should read (30, 31)

449-450- explain ‘lack of peer mentality’ briefly, was this observed / felt by interviewees?

451 – to clarify your point, change “some” to “further” as you’ve already mentioned a potential limitation in line 450.

455 – to clarify your point, change “provided further points of view” to “offered alternative or differing points of view.”.

456-457 – Qualitative work isn’t about representativeness of all studied populations so I don’t think this limitation is necessary. I think you should finish on a positive / strength, i.e. contribution to the literature / field and where it could go in the future.

460 – expand upon point RE financial incentives, reiterate that this is a fault in the current policy / medical system.

References

517-521 - reference 9. Is in German rather than English

522-524 - reference 10. Is in German rather than English

531-532 – reference 15. Is in German rather than English

550-551 – add DOI - Buss L F, Spitzer D, Watson J C. Can I have blood tests to check everything is alright? BMJ 2023; 382 :e075728 doi:10.1136/bmj-2023-075728

560-561 – add doi

564-567 - reference 29. Is in German rather than English

Supplementary Information

COREQ Checklist

13. Clarify why this information hasn’t been included?

Thanks again and hope this is helpful!

**********

what does this mean?). If published, this will include your full peer review and any attached files.

If you choose “no”, your identity will remain anonymous but your review may still be made public.

**Do you want your identity to be public for this peer review?**  For information about this choice, including consent withdrawal, please see our Privacy Policy

Reviewer #1: No

Reviewer #2: **Yes:**  Jordan Van Godwin

**********

[NOTE: If reviewer comments were submitted as an attachment file, they will be attached to this email and accessible via the submission site. Please log into your account, locate the manuscript record, and check for the action link "View Attachments". If this link does not appear, there are no attachment files.]

While revising your submission, please upload your figure files to the Preflight Analysis and Conversion Engine (PACE) digital diagnostic tool, https://pacev2.apexcovantage.com/. PACE helps ensure that figures meet PLOS requirements. To use PACE, you must first register as a user. Registration is free. Then, login and navigate to the UPLOAD tab, where you will find detailed instructions on how to use the tool. If you encounter any issues or have any questions when using PACE, please email PLOS at figures@plos.org

# Author comment: Author response to Decision Letter 1

**DOI:** 10.1371/journal.pone.0348530.r002

10 Dec 2025

PONE-D-25-32189

Monitoring routines for people with chronic conditions: Motivations, experiences and perspectives of physicians from different specialties - a qualitative interview study

PLOS ONE

Dear Editor,

Thank you very much for your consideration of our manuscript and for the opportunity to revise it. We greatly appreciate the constructive feedback provided by you and the reviewers, which helped us to improve the clarity, rigor, and overall quality of our work. In the following, we have addressed each comment and suggestion in detail and made corresponding revisions to the manuscript.

Yours sincerely,

Julia Heisig

1. Thank you very much for your support. To meet PLOS ONE's style requirements, we changed the figure and table naming, and updated any references in the main text. We also changed the formatting according to the provided guidelines.

2. We erased the non-matching funding statement from the article. Thank you for the clarification and your offer to change the funding statement in the online submission form. The accurate funding information is as follows:

“The research presented in this article was part of the project ChroMo (Monitoring routines for people with chronic diseases: Assessment and roadmap for the future) and was funded by the German Innovation Fund of the Federal Joint Committee (G-BA) [grant number 01VSF22046]. The funders had no role in study design, data collection and analysis, decision to publish, or preparation of the manuscript.”

3. We erased the funding concerning the publication funding by the DFG from the acknowledgement section. Also, this funding did not support the research for this article, only the publication process. The statement above is accurate (bullet point 2).

4. We have decided to publish the interview transcripts on zenodo to ensure long-term data availability: 10.5281/zenodo.17880631

Transcripts from our parallel interview study on patients’ perspective regarding ultrasound monitoring routines are also available on zenodo (https://doi.org/10.1371/journal.pone.0338431).

5. All supporting information were renamed in the article and were placed at the end of the manuscript according to the guideline. Captions are included at the end of the manuscript.

6. We removed all personal information from the supplementary table S3. The column “region in Germany” for each interviewed individual was erased as this information does not support full anonymization.

7. We added the following articles to our references. We reviewed them and found them relevant and appropriate to include:

12. Koschemann V, Warkentin L, Viniol A, Kühlein T, Hueber S, Werner F. Flying blind? Recommendations for monitoring of 10 common chronic diseases in guidelines from Germany, England, and Europe - a modified systematic review. BMC Med. 2025;23:671. doi:10.1186/s12916-025-04507-y.

13. Douglas PS, Garcia MJ, Haines DE, Lai WW, Manning WJ, Patel AR, et al. ACCF/ASE/AHA/ASNC/HFSA/HRS/SCAI/SCCM/SCCT/SCMR 2011 Appropriate Use Criteria for Echocardiography. A Report of the American College of Cardiology Foundation Appropriate Use Criteria Task Force, American Society of Echocardiography, American Heart Association, American Society of Nuclear Cardiology, Heart Failure Society of America, Heart Rhythm Society, Society for Cardiovascular Angiography and Interventions, Society of Critical Care Medicine, Society of Cardiovascular Computed Tomography, Society for Cardiovascular Magnetic Resonance American College of Chest Physicians. J Am Soc Echocardiogr. 2011;24:229–67. doi:10.1016/j.echo.2010.12.008.

14. Mader FM, Schwenke R. S3-Leitlinie Schlaganfall. DEGAM. 2020.

15. Hamann G, Sander D, Röther J, Grau A. Sekundärprophylaxe ischämischer Schlaganfall und transitorisch ischämische Attacke: Teil 1, S2k-Leitlinie. 2022.

16. Eckstein H-H, Kühnl A, Berkefeld J, Dörfler A, Kopp I, Langhoff R, et al., editors. S3-Leitlinie zur Diagnostik, Therapie und Nachsorge der extracraniellen Carotisstenose. AWMF. 2020.21. Malterud K, Siersma VD, Guassora AD. Sample Size in Qualitative Interview Studies: Guided by Information Power. Qual Health Res. 2016;26:1753–60. doi:10.1177/1049732315617444.

25. Braun V, Clarke V. Reflecting on reflexive thematic analysis. Qualitative Research in Sport, Exercise and Health. 2019;11:589–97. doi:10.1080/2159676X.2019.1628806.

26. Braun V, Clarke V. Thematic Analysis: A Practical Guide. 1st ed.: SAGE Publications Ltd; 2021.8.

8. We changed the citation style and reference list to Vancouver (Springer) to correspond to the journal’s guidelines.

# Peer review: Decision Letter 1

**DOI:** 10.1371/journal.pone.0348530.r003

**Authors:** Ahmed Qasim Mohammed Alhatemi

27 Jan 2026

Dear Dr. Heisig,

Please submit your revised manuscript by Mar 13 2026 11:59PM. If you will need more time than this to complete your revisions, please reply to this message or contact the journal office at plosone@plos.org. When you're ready to submit your revision, log on to https://www.editorialmanager.com/pone/ and select the 'Submissions Needing Revision' folder to locate your manuscript file.

- A letter that responds to each point raised by the academic editor and reviewer(s). You should upload this letter as a separate file labeled 'Response to Reviewers'.
- A marked-up copy of your manuscript that highlights changes made to the original version. You should upload this as a separate file labeled 'Revised Manuscript with Track Changes'.
- An unmarked version of your revised paper without tracked changes. You should upload this as a separate file labeled 'Manuscript'.

If applicable, we recommend that you deposit your laboratory protocols in protocols.io to enhance the reproducibility of your results. Protocols.io assigns your protocol its own identifier (DOI) so that it can be cited independently in the future. For instructions see: https://journals.plos.org/plosone/s/submission-guidelines#loc-laboratory-protocols. Additionally, PLOS ONE offers an option for publishing peer-reviewed Lab Protocol articles, which describe protocols hosted on protocols.io. Read more information on sharing protocols at https://plos.org/protocols?utm_medium=editorial-email&utm_source=authorletters&utm_campaign=protocols.

We look forward to receiving your revised manuscript.

Kind regards,

Ahmed Qasim Mohammed Alhatemi, Mbchb

Academic Editor

PLOS One

Journal Requirements:

If the reviewer comments include a recommendation to cite specific previously published works, please review and evaluate these publications to determine whether they are relevant and should be cited. There is no requirement to cite these works unless the editor has indicated otherwise.

Please review your reference list to ensure that it is complete and correct. If you have cited papers that have been retracted, please include the rationale for doing so in the manuscript text, or remove these references and replace them with relevant current references. Any changes to the reference list should be mentioned in the rebuttal letter that accompanies your revised manuscript. If you need to cite a retracted article, indicate the article’s retracted status in the References list and also include a citation and full reference for the retraction notice.

[Note: HTML markup is below. Please do not edit.]

Reviewers' comments:

Reviewer's Responses to Questions

**Comments to the Author**

Reviewer #2: All comments have been addressed

**********

2. Is the manuscript technically sound, and do the data support the conclusions?

Reviewer #2: Yes

**********

3. Has the statistical analysis been performed appropriately and rigorously?

Reviewer #2: Yes

**********

4. Have the authors made all data underlying the findings in their manuscript fully available?

The PLOS Data policy

Reviewer #2: Yes

**********

5. Is the manuscript presented in an intelligible fashion and written in standard English?

Reviewer #2: Yes

**********

Reviewer #2: Thank you for the opportunity to read your paper again. The revisions have enhanced the clarity and contribution of your work.

A couple of small further change suggested,

Abstract Line 32-33 - add appropriate reference to Braun and Clarke reference "Transcripts were analysed with thematic analysis according to Braun and Clarke (REFERENCE HERE)".

Methods Line 174 - 174 – to ensure consistency with how Braun and Clarke describe their form analysis change "reflexible thematic analysis" to "reflexive thematic analysis".

All the best,

Jordan

**********

what does this mean?). If published, this will include your full peer review and any attached files.

If you choose “no”, your identity will remain anonymous but your review may still be made public.

**Do you want your identity to be public for this peer review?**  For information about this choice, including consent withdrawal, please see our Privacy Policy

Reviewer #2: **Yes:**  Jordan Van Godwin

**********

[NOTE: If reviewer comments were submitted as an attachment file, they will be attached to this email and accessible via the submission site. Please log into your account, locate the manuscript record, and check for the action link "View Attachments". If this link does not appear, there are no attachment files.]

To ensure your figures meet our technical requirements, please review our figure guidelines: https://journals.plos.org/plosone/s/figures

You may also use PLOS’s free figure tool, NAAS, to help you prepare publication quality figures: https://journals.plos.org/plosone/s/figures#loc-tools-for-figure-preparation.

NAAS will assess whether your figures meet our technical requirements by comparing each figure against our figure specifications.

# Author comment: Author response to Decision Letter 2

**DOI:** 10.1371/journal.pone.0348530.r004

16 Feb 2026

Dear Reviewers,

Thank you very much for the opportunity to revise our manuscript. We greatly appreciate the constructive feedback and believe that the review process has further improved the quality and clarity of our work.

We have addressed both comments in this second review round. Please find our detailed responses below. All corresponding changes in the manuscript are marked in yellow.

In addition, a few minor technical corrections occurred during revision due to the automated citation tool (lines 52 and 164). These adjustments are also highlighted in yellow.

Thank you again for your time and consideration.

Sincerely,

Julia Heisig

Abstract Line 32-33 - add appropriate reference to Braun and Clarke reference "Transcripts were analysed with thematic analysis according to Braun and Clarke (REFERENCE HERE)".

Response: Thank you for this comment. According to the PLOS ONE guidelines, abstracts should not include references. Therefore, we did not add the Braun and Clarke citation in the abstract and instead kept the reference in the Methods section. If preferred by the editor, we would of course be happy to include it.

Methods Line 174 - 174 – to ensure consistency with how Braun and Clarke describe their form analysis change "reflexible thematic analysis" to "reflexive thematic analysis".

Response: Thank you very much for your careful reading of the manuscript and pointing out this error. We have corrected to “reflexive thematic analysis” accordingly (line 163).

# Peer review: Decision Letter 2

**DOI:** 10.1371/journal.pone.0348530.r005

**Authors:** Emma Campbell

6 May 2026

Dear Dr. Heisig,

Please submit your revised manuscript by Jun 19 2026 11:59PM. If you will need more time than this to complete your revisions, please reply to this message or contact the journal office at plosone@plos.org. When you're ready to submit your revision, log on to https://www.editorialmanager.com/pone/ and select the 'Submissions Needing Revision' folder to locate your manuscript file.

- A letter that responds to each point raised by the academic editor and reviewer(s). You should upload this letter as a separate file labeled 'Response to Reviewers'.
- A marked-up copy of your manuscript that highlights changes made to the original version. You should upload this as a separate file labeled 'Revised Manuscript with Track Changes'.
- An unmarked version of your revised paper without tracked changes. You should upload this as a separate file labeled 'Manuscript'.

If you would like to make changes to your financial disclosure, please include your updated statement in your cover letter. Guidelines for resubmitting your figure files are available below the reviewer comments at the end of this letter.

If applicable, we recommend that you deposit your laboratory protocols in protocols.io to enhance the reproducibility of your results. Protocols.io assigns your protocol its own identifier (DOI) so that it can be cited independently in the future. For instructions see: https://journals.plos.org/plosone/s/submission-guidelines#loc-laboratory-protocols. Additionally, PLOS ONE offers an option for publishing peer-reviewed Lab Protocol articles, which describe protocols hosted on protocols.io. Read more information on sharing protocols at https://plos.org/protocols?utm_medium=editorial-email&utm_source=authorletters&utm_campaign=protocols.

As the corresponding author, your ORCID iD is verified in the submission system and will appear in the published article. PLOS supports the use of ORCID, and we encourage all coauthors to register for an ORCID iD and use it as well. Please encourage your coauthors to verify their ORCID iD within the submission system before final acceptance, as unverified ORCID iDs will not appear in the published article. *Only*  the individual author can complete the verification step; PLOS staff *cannot*  verify ORCID iDs on behalf of authors.

We look forward to receiving your revised manuscript.

Kind regards,

Emma Campbell, Ph.D

Staff Editor

PLOS One

Journal Requirements:

If the reviewer comments include a recommendation to cite specific previously published works, please review and evaluate these publications to determine whether they are relevant and should be cited. There is no requirement to cite these works unless the editor has indicated otherwise.

[Note: HTML markup is below. Please do not edit.]

Reviewers' comments:

Reviewer's Responses to Questions

**Comments to the Author**

Reviewer #3: (No Response)

Reviewer #4: (No Response)

**********

2. Is the manuscript technically sound, and do the data support the conclusions?

Reviewer #3: Yes

Reviewer #4: Partly

**********

3. Has the statistical analysis been performed appropriately and rigorously?

Reviewer #3: Yes

Reviewer #4: N/A

**********

4. Have the authors made all data underlying the findings in their manuscript fully available?

The PLOS Data policy

Reviewer #3: Yes

Reviewer #4: Yes

**********

5. Is the manuscript presented in an intelligible fashion and written in standard English?

Reviewer #3: Yes

Reviewer #4: Yes

**********

Reviewer #3: The manuscript addresses a sensitive and clinically important issue from the perspectives of patients, clinicians, and the healthcare system. The study is methodologically sound: the design is appropriate to the research objectives, and the methods are clearly described and justified. The manuscript is well written and easy to follow, and the inclusion of direct quotations from interviewees enhances transparency and facilitates a deeper understanding of the findings.

The authors provide a thorough comparison of their results with the existing literature and present a balanced discussion of the study’s strengths and limitations. The Conclusions section appropriately highlights key questions emerging from the findings and underscores the need for further research, particularly to evaluate current monitoring practices and to integrate evidence-based recommendations into clinical guidelines aimed at optimizing patient outcomes and resource utilization.

To further strengthen the manuscript, it would be valuable for the authors to articulate more explicitly the novel contribution of this study to the existing body of knowledge. A clearer statement of what specifically advances current understanding would enhance the overall impact and relevance of the work.

Reviewer #4: Monitoring routines for people with chronic conditions: Motivations, experiences and perspectives of physicians from different specialties - a qualitative interview study

By Heisig et. al.

The paper concerns interviews over the relevance and timeliness of monitoring routines for chronic heart disease (CHD) and stroke. My immediate impression is that the paper is relevant, with the survey appropriately carried out, and, to some extent, appropriately summarized. There are however in my opinion a number of things that the authors should address prior to publication. In particular the discussion section appears weak, and would benefit from describing the results and their implications in light of existing literature, rather than the so-called 'comparison with existing literature' that is currently the main section of the discussion. Furthermore it appears to me that some of the conclusions are too strict from the presented evidence. Specific things to attend are listed below.

A. The title. The title indicate general monitoring routines for 'chronic conditions'. But this is not what the authors investigate. 'Chronic diseases' are limited to two diseases, CHD and stroke, which are both diseases from the circulary system, and the authors have not justified nor discussed overlap with general chronic diseases in the monitoring routines. This should be reflected in the title.

B. How have cardiologists and neurologists been selected? The text only describe how they are contacted (email found through web page), but not why the individual cariologists/neurologists were contacted.

C. The structure of the 'Data analysis' section should be tightened. The authors decide to describe 6 steps in a reference to a thematic approach to qualitative analysis. Steps 1-3 and 5 are numbered in the description, but step 4 is referred to as 'the next step' and the contents of step 6 is not specified. Please tighten up this notation.

D. The interviews are grouped to GPs, cardiologists and neurologists. However, when going over the results, it appears that in particular neurologists are not mentioned. For example, in the 'Subtheme: DMPs and guidelines as framework', the GP attitude and cardiologist attitudes are scrutinized, but neurologists are not mentioned. Despite what seems to be a quote from a neurologist (N-19). Attitudes should be described either as a general trend with specification of deviations among the groups, or be mentioning all three groups. Otherwise the results are partial.

E. In light of the results in Theme 4, I will question whether the authors have evidence for writing that the regular use of EKG/ultrasound was considered unnecessary. Theme 4 lists benefits from monitoring with EKC/ultrasound, and while other themes may indicate attitudes that support that such monitoring is unnecessary, this may be voiced at other interviews than those that lists benefits in Theme 4, essentially dividing the sample population into two heterogeneous groups, where one group voice benefits and the other that it is unnecessary. The authors should argue from their thematic approach that this is not the case, if the evaluation of the EKG/ultrasound as unnecessary is maintained.

F. The use of past tense in the section 'comparison with existing literature'. Here it is difficult to distinguish when the authors refer to results from the literature, and when it is aspects of their own results that is discussed. In terms of what the comparison means prospectively, it is odd that past tense is used. For example lines 464-467, where it is (was) essential with continuous medical education and evaluation of diagnostic technologies.

G. I am curious as to why patients perception of intervention benefits is discussed in lines 445-448 as it is. There are no patients in this study, only the thoughts from the physicians about such, which may be a very different thing. The discussion should refflect that.

H. I believe that the authors in a number of passages are overstating the evidence from the survey. In line 449, they mention 'overtesting'; but do their data data support that overtesting takes place, whether overtesting is the rule? And what do the authors consider as 'overtesting'? Needless to say, more testing than needed, but when is the benefit sufficiently small to rate a test procedure as 'overtesting'?

I. In the S&L section, the authors claim that the work captures 'perspectives that extend beyond the viewpoint of any single speciality'. Considering that the authors only look into 2 specialities, which are even for the same group of diseases, I think that such a claim is stretching it a bit, cf. my view on the title of the paper. The authors should argue for that the information gathered can be formulated in such a broad way. It is worth noting that the authors inline 499 state that 'physicians from other specialities also could have additional perspectives'. Yes indeed.

J. In the conclusion I believe that the authors overstate the evidence from the survey. For example, they write that 'The medical benefits of ultrasound are limited for CHD and stroke patients'. The authors cannot know that. The evidence is at the most that the benefit APPEARS limited from the survey, but to draw a conclusion beyond the material, and indeed over all interviewees, as as I see it unfounded.

K. In the abstract, the authors state that 'transcripts were analysed with thematic analysis according to Braun and Clarke'. 'Braun and Clarke' is here the authors of a publication that the authors refer to wrt. thematic analysis. However, in the abstract, these names should not appear, thematic analysis in itself should be enough, as the reference may be found in the main text. Laypersons may be mislead to think that 'Braun and Clarke' is a principle rather than a reference, which there by the way is two of (two papers of Braun and Clarke).

L. Grammar: Line 47, line 130.

**********

what does this mean?). If published, this will include your full peer review and any attached files.

If you choose “no”, your identity will remain anonymous but your review may still be made public.

**Do you want your identity to be public for this peer review?**  For information about this choice, including consent withdrawal, please see our Privacy Policy

Reviewer #3: **Yes:**  Marine Gambaryan, MD, MPH, D.M.Sc, Associate Professor

Reviewer #4: **Yes:**  Anders Stockmarr

**********

[NOTE: If reviewer comments were submitted as an attachment file, they will be attached to this email and accessible via the submission site. Please log into your account, locate the manuscript record, and check for the action link "View Attachments". If this link does not appear, there are no attachment files.]

To ensure your figures meet our technical requirements, please review our figure guidelines: https://journals.plos.org/plosone/s/figures

You may also use PLOS’s free figure tool, NAAS, to help you prepare publication quality figures: https://journals.plos.org/plosone/s/figures#loc-tools-for-figure-preparation.

NAAS will assess whether your figures meet our technical requirements by comparing each figure against our figure specifications.

# Author comment: Author response to Decision Letter 3

**DOI:** 10.1371/journal.pone.0348530.r006

22 Jun 2026

Dear Reviewers,

thank you very much for the revision of our manuscript. We greatly appreciate the constructive feedback which helped us to improve the clarity and quality of our work. In the following, we have addressed each comment and suggestion in detail and made corresponding revisions to the manuscript. All line numbers refer to the manuscript with track changes.

Yours sincerely,

Julia Heisig

Reviewer #3:

The manuscript addresses a sensitive and clinically important issue from the perspectives of patients, clinicians, and the healthcare system. The study is methodologically sound: the design is appropriate to the research objectives, and the methods are clearly described and justified. The manuscript is well written and easy to follow, and the inclusion of direct quotations from interviewees enhances transparency and facilitates a deeper understanding of the findings.

The authors provide a thorough comparison of their results with the existing literature and present a balanced discussion of the study’s strengths and limitations. The Conclusions section appropriately highlights key questions emerging from the findings and underscores the need for further research, particularly to evaluate current monitoring practices and to integrate evidence-based recommendations into clinical guidelines aimed at optimizing patient outcomes and resource utilization.

To further strengthen the manuscript, it would be valuable for the authors to articulate more explicitly the novel contribution of this study to the existing body of knowledge. A clearer statement of what specifically advances current understanding would enhance the overall impact and relevance of the work.

Response: We thank the reviewer for this important suggestion. We have now added a clearer statement in the Introduction to explicitly highlight the novel contribution of our study (lines 97-103).

Building on previous work showing that many monitoring routines in chronic conditions lack a strong evidence base, our study adds a multi-perspective explanation of why such routines are still performed in clinical practice. Specifically, within the ChroMo project we combine physicians’ interviews (this manuscript), patients’ perspectives, and complementary routine data to explore motivations and perceived benefits of monitoring routines across different specialties and two diseases.

This paper specifically contributes an in-depth understanding of physicians’ perspectives on why routine imaging monitoring may continue in clinical practice despite limited evidence. By including GPs, cardiologists and neurologists, and by focusing on two clinical examples, coronary heart disease and post-stroke care, the study highlights how clinical reasoning, perceived patient needs, safety concerns, collaboration patterns and reimbursement structures interact in everyday monitoring practices.

Reviewer #4:

Monitoring routines for people with chronic conditions: Motivations, experiences and perspectives of physicians from different specialties - a qualitative interview study

By Heisig et. al.

The paper concerns interviews over the relevance and timeliness of monitoring routines for chronic heart disease (CHD) and stroke. My immediate impression is that the paper is relevant, with the survey appropriately carried out, and, to some extent, appropriately summarized. There are however in my opinion a number of things that the authors should address prior to publication. In particular the discussion section appears weak, and would benefit from describing the results and their implications in light of existing literature, rather than the so-called 'comparison with existing literature' that is currently the main section of the discussion. Furthermore it appears to me that some of the conclusions are too strict from the presented evidence. Specific things to attend are listed below.

A. The title. The title indicate general monitoring routines for 'chronic conditions'. But this is not what the authors investigate. 'Chronic diseases' are limited to two diseases, CHD and stroke, which are both diseases from the circulary system, and the authors have not justified nor discussed overlap with general chronic diseases in the monitoring routines. This should be reflected in the title.

Response: Thank you for your feedback. We agree with your reasoning and changed the title accordingly. We removed the expression “chronic conditions” and stated the two diseases we investigated.

Revised full title:

Motivations, experiences and perspectives of physicians in Germany regarding monitoring routines in coronary heart disease and post-stroke patients: a qualitative interview study

Revised short title:

Physicians’ views on monitoring routines in coronary heart disease and post-stroke patients

B. How have cardiologists and neurologists been selected? The text only describe how they are contacted (email found through web page), but not why the individual cariologists/neurologists were contacted.

Response: We added more details to our recruitment strategy to make it clearer how cardiologists and neurologists were recruited. We added the following paragraph (line 126):

Cardiologists and neurologists from the local region were initially approached; if no response was received within a few days, recruitment was expanded to a broader geographic area within Northern- and Middle-Hesse and Northern Bavaria. Recruitment efforts were finalised when all participants appeared well-balanced regarding practice setting (region, rural/urban and single/joint), specialty, age and practical experience.

C. The structure of the 'Data analysis' section should be tightened. The authors decide to describe 6 steps in a reference to a thematic approach to qualitative analysis. Steps 1-3 and 5 are numbered in the description, but step 4 is referred to as 'the next step' and the contents of step 6 is not specified. Please tighten up this notation.

Response: We added the numeration of each step in lines 190 and 195.

D. The interviews are grouped to GPs, cardiologists and neurologists. However, when going over the results, it appears that in particular neurologists are not mentioned. For example, in the 'Subtheme: DMPs and guidelines as framework', the GP attitude and cardiologist attitudes are scrutinized, but neurologists are not mentioned. Despite what seems to be a quote from a neurologist (N-19). Attitudes should be described either as a general trend with specification of deviations among the groups, or be mentioning all three groups. Otherwise the results are partial.

Response: We added a paragraph to describe the neurologists‘ view (line 255). Neurologists found guidelines in general important, but guidelines either lack specific working instructions or neurologist are unsure what they say due to not reading them for a long time.

In the following themes we did not specify between cardiologists and neurologists, whenever their views overlap and could be combined. There was no benefit to distinguish between these two specialties and we refer to both as “physicians” (often together with GPs).

The paragraph in line 253 made sense to add as there was a difference between cardiologists and neurologists in how they use guidelines. Thank you for pointing this out.

E. In light of the results in Theme 4, I will question whether the authors have evidence for writing that the regular use of EKG/ultrasound was considered unnecessary. Theme 4 lists benefits from monitoring with EKC/ultrasound, and while other themes may indicate attitudes that support that such monitoring is unnecessary, this may be voiced at other interviews than those that lists benefits in Theme 4, essentially dividing the sample population into two heterogeneous groups, where one group voice benefits and the other that it is unnecessary. The authors should argue from their thematic approach that this is not the case, if the evaluation of the EKG/ultrasound as unnecessary is maintained.

Response: We agree with the reviewer that the term “unnecessary” does not adequately reflect the complexity of the findings. The interviewed physicians said that monitoring of asymptomatic CHD and post-stroke patients has benefits but these benefits are not directly connected to medical consequences of these examinations. The benefits are overarching like meeting their patients need for safety or their own need for assurance to not miss anything. An additional driving force for monitoring routines are the financial incentives. The medical necessity of these examinations is subordinated. But we also agree that “unnecessary” does not fit and should be avoided as it does not reflect the complexity of what are the driving forces behind monitoring routines.

We adjusted the wording in line 454 and 507 to avoid or weaken the expression “unnecessary”. We agree that it now better reflects the physicians’ views.

F. The use of past tense in the section 'comparison with existing literature'. Here it is difficult to distinguish when the authors refer to results from the literature, and when it is aspects of their own results that is discussed. In terms of what the comparison means prospectively, it is odd that past tense is used. For example lines 464-467, where it is (was) essential with continuous medical education and evaluation of diagnostic technologies.

Response: The whole discussion section was revised and the use of simple present and past tense was corrected.

G. I am curious as to why patients perception of intervention benefits is discussed in lines 445-448 as it is. There are no patients in this study, only the thoughts from the physicians about such, which may be a very different thing. The discussion should refflect that.

Response: We agree that the present study reports physicians’ perspectives and does not directly investigate patients’ views. However, we feel it is important to emphasize that the physicians’ views about their patients were also seen in the patients’ perspective. Especially, as both perspectives are investigated in the ChroMo study: the physicians’ and patients’ interview study are both sub projects of the ChroMo study. The recited paper [17] refers to the interview study of patients, conducted by our group at the same time. We added more explanation as to why we included the patients’ views in the discussion (line 461). The following sentence describes the reasons why monitoring is important for patients that was also described in our patients’ article and other studies.

Note: the reference given in brackets (Rink et al. 2025, under review) was removed and incorporated as a citation [17], as the article has been recently published.

H. I believe that the authors in a number of passages are overstating the evidence from the survey. In line 449, they mention 'overtesting'; but do their data data support that overtesting takes place, whether overtesting is the rule? And what do the authors consider as 'overtesting'? Needless to say, more testing than needed, but when is the benefit sufficiently small to rate a test procedure as 'overtesting'?

Response: Thank you for pointing this out. We appreciate your concern regarding the use of the term "overtesting." Some interviewed physicians used this term directly (not cited), and we believe the use of this word reflects the perspective from our interviews.

We agree that we should avoid overstating our findings. We used the term only in the discussion section when reflecting our results in the context of other articles that investigated overtesting, as their findings showed parallels to our results.

We revised the wording (lines 457, 469) and now used the term only when reflecting the results of other studies.

I. In the S&L section, the authors claim that the work captures 'perspectives that extend beyond the viewpoint of any single speciality'. Considering that the authors only look into 2 specialities, which are even for the same group of diseases, I think that such a claim is stretching it a bit, cf. my view on the title of the paper. The authors should argue for that the information gathered can be formulated in such a broad way. It is worth noting that the authors inline 499 state that 'physicians from other specialities also could have additional perspectives'. Yes indeed.

Response: We agree that the original wording may have overstated the breadth of perspectives captured in our study. Therefore, we revised the sentence to a more cautious formulation and now state that the study captured perspectives beyond those of a single specialty (line 501).

We would also like to clarify that the study included physicians from three specialties: general medicine, cardiology, and neurology. In Germany, general medicine is a medical specialty with a structured vocational training program comparable in duration and organization to those of cardiology and neurology. Nevertheless, we agree that physicians from other specialties may have additional perspectives, as discussed in the limitations section.

J. In the conclusion I believe that the authors overstate the evidence from the survey. For example, they write that 'The medical benefits of ultrasound are limited for CHD and stroke patients'. The authors cannot know that. The evidence is at the most that the benefit APPEARS limited from the survey, but to draw a conclusion beyond the material, and indeed over all interviewees, as as I see it unfounded.

Response: We agree that this sentence overstated our findings. We therefore revised the wording to weaken the statement and specified that it refers to asymptomatic patients. The sentence now reads:

The medical benefits of ultrasounds appear limited for asymptomatic CHD and stroke patients (line 526).

K. In the abstract, the authors state that 'transcripts were analysed with thematic analysis according to Braun and Clarke'. 'Braun and Clarke' is here the authors of a publication that the authors refer to wrt. thematic analysis. However, in the abstract, these names should not appear, thematic analysis in itself should be enough, as the reference may be found in the main text. Laypersons may be mislead to think that 'Braun and Clarke' is a principle rather than a reference, which there by the way is two of (two papers of Braun and Clarke).

Response: We removed the authors “Braun and Clarke” as we agree that it is not necessary to mention authors in an abstract (line 32).

L. Grammar: Line 47, line 130.

Response: Thank you for your helpful input, we corrected both errors accordingly (now line 51 and 145).

Attachment Submitted filename: Response to Reviewers_2026-06-19.docx

# Peer review: Decision Letter 3

**DOI:** 10.1371/journal.pone.0348530.r007

**Authors:** Johanna Pruller

3 Aug 2026

Motivations, experiences and perspectives of physicians in Germany regarding monitoring routines in coronary heart disease and post-stroke patients: a qualitative interview study

PONE-D-25-32189R3

Dear Dr. Heisig,

We’re pleased to inform you that your manuscript has been judged scientifically suitable for publication and will be formally accepted for publication once it meets all outstanding technical requirements.

Within one week, you’ll receive an e-mail detailing the required amendments. When these have been addressed, you’ll receive a formal acceptance letter and your manuscript will be scheduled for publication.

An invoice will be generated when your article is formally accepted. Please note, if your institution has a publishing partnership with PLOS and your article meets the relevant criteria, all or part of your publication costs will be covered. Please make sure your user information is up-to-date by logging into Editorial Manager at Editorial Manager® and clicking the ‘Update My Information' link at the top of the page. For questions related to billing, please contact billing support.

If your institution or institutions have a press office, please notify them about your upcoming paper to help maximize its impact. If they’ll be preparing press materials, please inform our press team as soon as possible -- no later than 48 hours after receiving the formal acceptance. Your manuscript will remain under strict press embargo until 2 pm Eastern Time on the date of publication. For more information, please contact onepress@plos.org.

Kind regards,

Johanna Pruller, Ph.D.

Senior Editor

PLOS One

Additional Editor Comments (optional):

Reviewers' comments:

Reviewer's Responses to Questions

**Comments to the Author**

Reviewer #4: All comments have been addressed

**********

2. Is the manuscript technically sound, and do the data support the conclusions?

Reviewer #4: Yes

**********

3. Has the statistical analysis been performed appropriately and rigorously?

Reviewer #4: N/A

**********

4. Have the authors made all data underlying the findings in their manuscript fully available?

The PLOS Data policy

Reviewer #4: Yes

**********

5. Is the manuscript presented in an intelligible fashion and written in standard English?

Reviewer #4: Yes

**********

Reviewer #4: The authors have addressed my concerns, the manuscript now appears publishable. I wish them the best in the further publication process.

**********

what does this mean?). If published, this will include your full peer review and any attached files.

If you choose “no”, your identity will remain anonymous but your review may still be made public.

**Do you want your identity to be public for this peer review?**  For information about this choice, including consent withdrawal, please see our Privacy Policy

Reviewer #4: **Yes:**  Anders Stockmarr

**********

# Editor report: Acceptance letter

**DOI:** 10.1371/journal.pone.0348530.r008

**Authors:** Johanna Pruller

PONE-D-25-32189R3

PLOS One

Dear Dr. Heisig,

I'm pleased to inform you that your manuscript has been deemed suitable for publication in PLOS One. Congratulations! Your manuscript is now being handed over to our production team.

At this stage, our production department will prepare your paper for publication. This includes ensuring the following:

* All references, tables, and figures are properly cited

* All relevant supporting information is included in the manuscript submission,

* There are no issues that prevent the paper from being properly typeset

You will receive further instructions from the production team, including instructions on how to review your proof when it is ready. Please keep in mind that we are working through a large volume of accepted articles, so please give us a few days to review your paper and let you know the next and final steps.

Lastly, if your institution or institutions have a press office, please let them know about your upcoming paper now to help maximize its impact. If they'll be preparing press materials, please inform our press team within the next 48 hours. Your manuscript will remain under strict press embargo until 2 pm Eastern Time on the date of publication. For more information, please contact onepress@plos.org.

You will receive an invoice from PLOS for your publication fee after your manuscript has reached the completed accept phase. If you receive an email requesting payment before acceptance or for any other service, this may be a phishing scheme. Learn how to identify phishing emails and protect your accounts at https://explore.plos.org/phishing.

If we can help with anything else, please email us at customercare@plos.org.

Thank you for submitting your work to PLOS One and supporting open access.

Kind regards,

PLOS One Editorial Office Staff

on behalf of

Dr. Johanna Pruller

Staff Editor

PLOS One
